# Long‐term ferrocyanide application via deicing salts promotes the establishment of Actinomycetales assimilating ferrocyanide‐derived carbon in soil

**DOI:** 10.1111/1751-7915.12362

**Published:** 2016-05-19

**Authors:** Silvia Gschwendtner, Tim Mansfeldt, Susanne Kublik, Evangelia Touliari, Franz Buegger, Michael Schloter

**Affiliations:** ^1^ Research Unit Environmental GenomicsHelmholtz Zentrum MünchenGerman Research Center for Environmental Health (GmbH)Ingolstädter Landstraße 1Neuherberg85764Germany; ^2^ Department Geowissenschaften, Bodengeographie/BodenkundeUniversität zu KölnAlbertus‐Magnus‐PlatzKöln50923Germany; ^3^ Institute of Biochemical Plant PathologyHelmholtz Zentrum MünchenGerman Research Center for Environmental Health (GmbH)Ingolstädter Landstraße 1Neuherberg85764Germany

## Abstract

Cyanides are highly toxic and produced by various microorganisms as defence strategy or to increase their competitiveness. As degradation is the most efficient way of detoxification, some microbes developed the capability to use cyanides as carbon and nitrogen source. However, it is not clear if this potential also helps to lower cyanide concentrations in roadside soils where deicing salt application leads to significant inputs of ferrocyanide. The question remains if biodegradation in soils can occur without previous photolysis. By conducting a microcosm experiment using soils with/without pre‐exposition to road salts spiked with ^13^C‐labelled ferrocyanide, we were able to confirm biodegradation and in parallel to identify bacteria using ferrocyanide as C source via DNA stable isotope probing (DNA‐SIP), TRFLP fingerprinting and pyrosequencing. Bacteria assimilating ^13^C were highly similar in the pre‐exposed soils, belonging mostly to *Actinomycetales* (*Kineosporia*,* Mycobacterium*,* Micromonosporaceae*). In the soil without pre‐exposition, bacteria belonging to Aci*dobacteria* (Gp3, Gp4, Gp6), *Gemmatimonadetes* (*Gemmatimonas*) and *Gammaproteobacteria* (*Thermomonas*,* Xanthomonadaceae*) used ferrocyanide as C source but not the present *Actinomycetales*. This indicated that (i) various bacteria are able to assimilate ferrocyanide‐derived C and (ii) long‐term exposition to ferrocyanide applied with deicing salts leads to *Actinomycetales* outcompeting other microorganisms for the use of ferrocyanide as C source.

## Introduction

Cyanide is produced by various organisms including bacteria, algae, fungi and higher plants as defence mechanism or offensive strategy (Møller and Seigler, 1998; Gallagher and Manoil, 2001; Zagrobelny *et al*., 2008) and consequently occurs naturally at low levels in the environment. However, it is highly toxic for living organisms by complexing metalloproteins, e.g. cytochrome oxidase, and thus inhibiting protein function (Solomonson, 1981). Besides, cyanide severs nutrient limitation in the environment by the formation of very stable metal‐cyanide complexes making essential nutrients unavailable to organisms. Thus, organisms growing in the presence of cyanide must have an alternative cyanide‐insensitive metabolism (Jünemann, 1997; Berthold *et al*., 2000; Richardson, 2000) and/or the capacity to degrade cyanide to harmful molecules that can be used as C and N source. As the latter offers an additional advantage in nutrient limited environments, the capacity for cyanide biodegradation is widespread among soil microorganisms (Dubey and Holmes, 1995) and can occur via four pathways: (i) via hydrolysis, generating either formamide or formate and ammonia, (ii) via oxidative degradation, generating CO_2_ and ammonia, (iii) via reduction, generating methane and ammonia and (iv) via substitution, producing thiocyanate (Raybuck, 1992). It is postulated that in various environments close ‘microcycles’ of cyanogenic organisms and organisms assimilating cyanide as C and N source exist, depending on species composition and distribution (Allen and Strobel, 1966; Thatcher and Weaver, 1976).

In contrast to naturally occurring cyanide, anthropogenic activities produce large amounts of cyanide‐containing wastes entering the environment via disposal of gas‐purifier wastes from former manufactured gas or coke‐oven plants (Mansfeldt *et al*., 1998; Wehrer *et al*., 2011), landfilling of blast‐furnace and sewage sludge (Mansfeldt and Dohrmann, 2001; Rennert *et al*., 2007), soil amendment with paper de‐inking sludge (Mansfeldt, 2001) and waste water from mining and electroplating industry (Korte *et al*., 2000; Wong‐Chong *et al*., 2006). Most of the released cyanide is complexed with iron as ferrocyanide (Fe^II^(CN)_6_^4−^) or ferricyanide (Fe^III^(CN)_6_^3−^). Another significant release of metal‐cyanide complexes into the environment is the application of deicing salt containing ferrocyanide as anticaking agent (Paschka *et al*., 1999). In Germany, currently about 100 mg Fe(CN)_6_ kg^−1^ road salt are used, resulting in an annual input of 75–425 Mg cyanide to the road soil environment. However, the fate of ferrocyanide added via deicing salts into the environment remains unclear. Although iron‐cyanide complexes are very stable in the dark and thus have low toxicity in soil *per se*, they dissociate rapidly (8% h^−1^) when exposed to daylight (Meeussen *et al*., 1992), e.g. by transport of contaminated groundwater to the surface, releasing extremely toxic free cyanide. Several factors favour the adsorption (and consequently immobilization) of metal‐cyanide complexes to soil components, e.g. low pH, free iron oxide content, high clay content and high organic matter content (Alesii and Fuller, 1976; Sut *et al*., 2013). Coincidentally, Ohno (1990) found up to 200 μg total cyanide L^−1^ in adjacent surface waters (< 30 m) near uncovered salt storage facilities in Maine, USA. From simple soil sorption studies and the observation that measured cyanide surface water concentrations were lower than predicted ones, the authors concluded that adsorption had lowered cyanide concentrations in the soil during passage, indicating significant buffer capacity of soils. Despite its chemical stability, several studies reported microbial degradation of metal‐cyanide complexes by various soil microorganisms, using it as N and/or C source for growth (Aronstein *et al*., 1994; Barclay *et al*., 1998a,b; Dursun *et al*., 1999; Yanase *et al*., 2000; Kwon *et al*., 2002). Biodegradation was found to be mainly dependent on the physical nature of soils influencing substrate availability and on the availability of nutrients (especially C) and oxygen which is consumed during several cyanide degrading pathways (Raybuck, 1992; Dursun *et al*., 1999). As not all studies excluded photolysis of metal‐cyanide complexes prior to microbial degradation by their experimental setup or included controls to determine physical dissociation, it is difficult to decide whether the examined organisms used the metal‐cyanide complexes directly or whether biodegradation occurred solely upon dissociated free cyanide. Consequently, the question remains if the reported biodegradation of metal‐cyanides is more a function of the dissociation of the complex than of the microbial degradation.

Despite its ecological significance, data on the fate and biodegradation of ferrocyanide complexes in soils are missing so far. We hypothesize (i) that the microbial degradation will take place and that several soil microorganisms are able to use ferrocyanide as C source as this offers an additional advantage in nutrient limited environments. We postulate further (ii) that the microbial community in pre‐exposed roadside soils is adapted to ferrocyanide application, and consequently, microorganisms capable to assimilate ferrocyanide‐derived C are enriched when compared with a first exposed control soil. To test these hypotheses, we conducted a microcosm experiment using two roadside soils with pre‐exposition to deicing salt and a control soil with a comparable soil texture, which was collected in a wider distance to the road, thus not influenced by the deicing treatments. We used ^13^C‐labelled ferrocyanide and incubated the microcosms in the dark to prevent light‐mediated dissociation. By this we were able (i) to show whether biodegradation occurs and (ii) to identify microorganisms assimilating C derived from ferrocyanide complexes using DNA stable isotope probing (DNA‐SIP) combined with 16S rRNA amplicon‐based TRFLP fingerprinting and pyrosequencing. Our results revealed ^13^C incorporation into microbial biomass C as well as into DNA, indicating microbial degradation of the applied ferrocyanide. Although microbial community structure differed significantly among all soils, bacteria using ferrocyanide as C source were similar in the soils pre‐exposed to deicing salts but differed highly when compared with the soil without pre‐exposition. Detailed differences in microbial community composition and ^13^C assimilation among the soils are discussed.

## Results

### Microbial incorporation of ^13^C derived from ferrocyanide

Isotopic enrichment of microorganisms using ferrocyanide as additional C source for growth was performed in a microcosm experiment using soils from two roadsides (D, F) with pre‐exposition to deicing salts and one control soil (W) without pre‐exposition to deicing salts. All soils were spiked with K_4_[Fe(^13^CN)_6_] × 3 H_2_O and incubated over a period of 74 days. To confirm microbial assimilation of ^13^C, microbial biomass C (C_mic_) was extracted from soil samples after 18, 32 and 74 days of incubation. The δ^13^C values indicated an increasing enrichment up to 32 days with 70.5‰, 60.6‰ and 52.8‰ Vienna‐Pee Dee Belemnite (V‐PDB) for soil D, F and W, respectively, and remained constant hereafter (Table 1). For the identification of soil bacteria incorporating ^13^C derived from ferrocyanide, DNA‐SIP was used. To resolve ^13^C‐enriched DNA from the background of unlabelled DNA, DNA extracts were isopycnically centrifuged. Quantification of 16S rRNA genes in gradient fractions by real‐time PCR revealed an abundance shift towards fractions with higher buoyant density (BD) in soils spiked with ^13^C‐labelled ferrocyanide when compared with soils spiked with unlabelled ferrocyanide for the first time 32 days after application (Fig. S1), so this time point was selected for further analyses of the microbial community.

**Table 1:** ^13^C incorporation (‰ V‐PDB) into microbial biomass (C_mic_) of soil D, F and W derived from microcosms spiked with ^13^C‐labelled or unlabelled ferrocyanide after 18, 32 and 74 days of incubation (*n* = 3, sd means standard deviation)

δ^13^C C_mic_	‰ V‐PDB	D		F		W	
		Mean	sd	Mean	sd	Mean	sd
^12^C‐Cyanide	18d	−26.04	0.39	−26.40	3.19	−26.15	1.94
	32d	−28.73	1.47	−30.81	0.72	−28.68	1.27
	74d	−26.52	0.30	−28.16	0.28	−27.28	0.20
^13^C‐Cyanide	18d	22.78	5.71	10.98	3.45	−0.88	1.33
	32d	70.48	7.63	60.58	10.97	52.83	4.14
	74d	75.36	8.26	67.59	13.92	57.22	6.66

### TRFLP fingerprinting of density‐resolved bacterial communities

**Figure 1:**
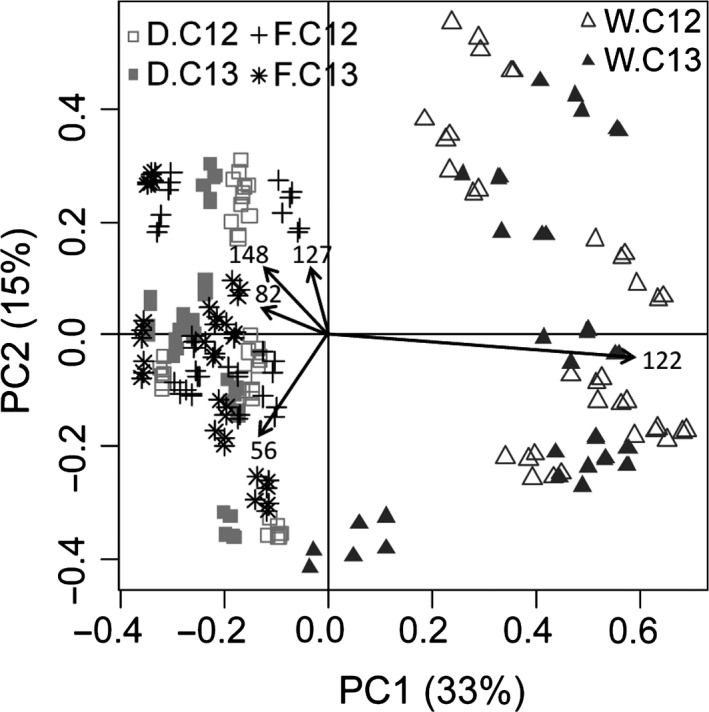
PCA plot generated from TRFLP fragments based on 16S rRNA gene amplicons from 12 gradient fractions of DNA directly extracted from soil D, F and W obtained from unlabelled (C12) and labelled (C13) microcosms (*n* = 3).

To compare microbial diversity among the soils, we reconstructed the total microbial soil communities by summarizing the abundance within the different fractions for each density gradient. In total, 108, 107 and 89 TRFs were detected for soils D, F and W, respectively, ranging from 53 to 802 bp 32 days after the application of the ferrocyanide. Although different regarding less abundant TRFs, soil D and F showed the same dominant TRFs; TRF 56 bp accounting for a relative abundance of 39% and 33%, respectively, followed by TRFs 148 bp, 82 bp and 127 bp which in sum accounted for 13% (D) and 9% (F) (Fig. 1, Table S1). Soil W differed significantly from the other soils, mainly due to the increased relative abundance of TRF 122 bp (48%) but decreased prevalence of TRF 56 bp (19%) (Fig. 1, Table S1). No significant influence of the ^13^C‐label on microbial community structure was observed when comparing the reconstructed microbial community of labelled and unlabelled density gradients (data not shown). Control microcosms without ferrocyanide application revealed a constant diversity pattern over the experimental period but significant differences between all three soils. Moreover, ferrocyanide application significantly affected microbial community composition in all soils, with most pronounced effect on soil W (data not shown).

**Figure 2:**
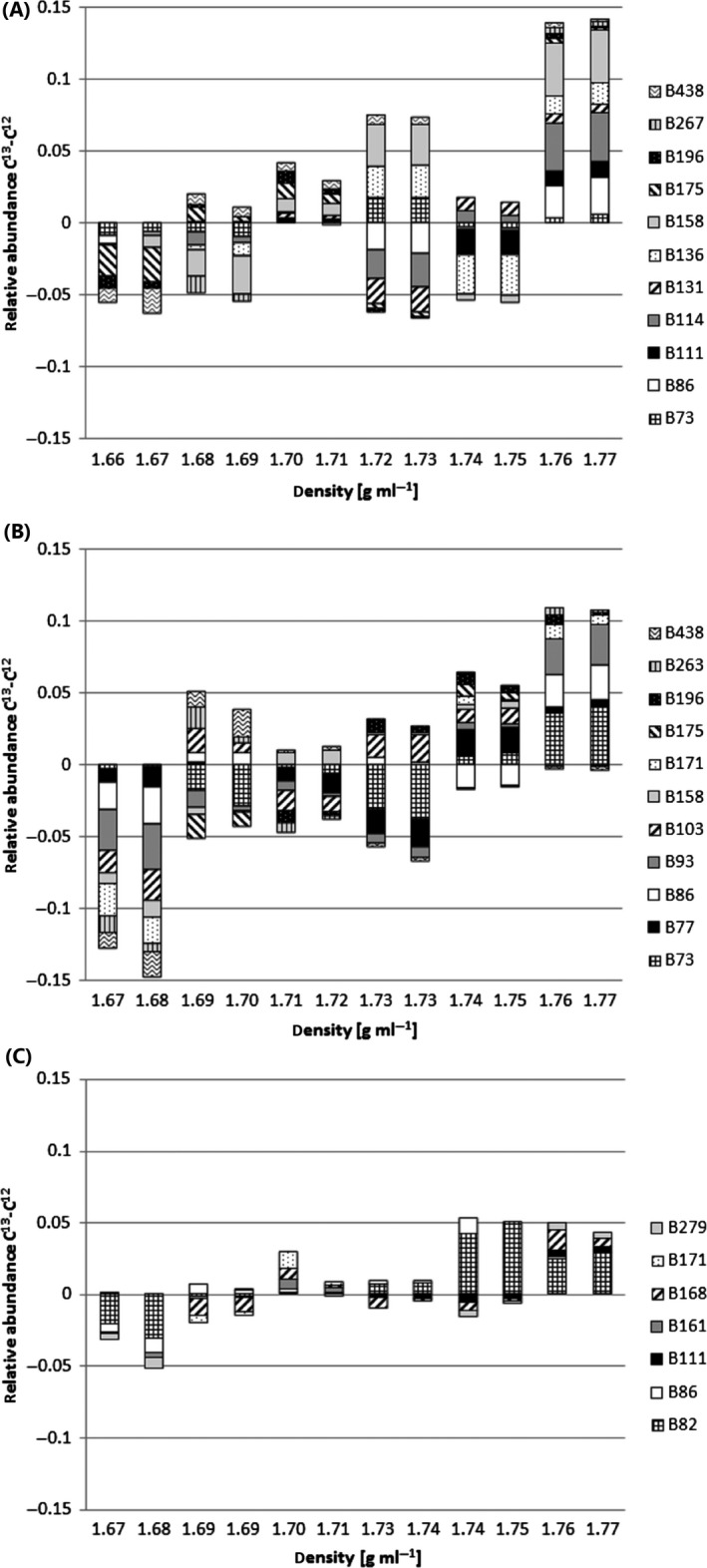
TRFLP fragments showing shifts towards fractions with higher buoyant density in ^13^C‐labelled microcosms when compared with unlabelled microcosms presented as mean difference between the relative abundance in ^13^C‐labelled and unlabelled DNA extracts from (A) soil D, (B) soil F and (C) soil W among 12 gradient fractions (*n* = 3). TRF name refers to TRF length in base pairs.

For the identification of TRFs representing bacteria assimilating ^13^C derived from ferrocyanide, the microbial diversity within the gradient fractions was screened for abundance shifts towards fractions with higher BD in ^13^C‐labelled soils when compared with soils spiked with unlabelled ferrocyanide. For both soil D and F, 11 TRFs were observed in ‘heavier’ fractions in the ^13^C gradient compared with the unlabelled gradient, from which the following were shifted in both soils: TRF 73, 158, 175, 196 and 438 bp (Fig. 2). TRFLP profiles of soil W gradients showed shifts for only 7 TRFs, from which TRF 111 bp was also identified as labelled in soil D but not F and TRF 171 bp was also shifted in soil F but not D (Fig. 2). TRF 86 bp was observed to be shifted towards ‘heavier’ fractions in all three soils. For all gradients, changes within the bacterial community structure occurred gradually over at least two gradient fractions. Consequently, the adjacent fractions showed quite similar TRFLP pattern (Fig. S2).

### Sequencing analysis of density‐resolved bacterial communities

Sample selection for 454 pyrosequencing analysis was done based on TRFLP results as it could be shown that TRFLP and 454 pyrosequencing are capable of recovering highly comparable community structure (Pilloni *et al*., 2012). As TRFLP pattern revealed similar microbial diversity in adjacent gradient fractions and high replicate similarity (Fig. S2), 36 samples were selected for 454 pyrosequencing analysis; every second gradient fraction (1, 3, 5, 7, 9, 11) of one randomly chosen sample for soil D, F and W, respectively, spiked with either ^13^C‐labelled or unlabelled ferrocyanide. In total, 350 086 bacterial raw sequence reads were generated from the 16S rRNA gene PCR amplicons. After filtering, chimera check and removing erroneous reads, 318 796 high‐quality sequence reads with a minimum length of 250 bp remained, which were represented by 22 165 operational taxonomic units (OTUs) at 97% sequence similarity. For subsampling of 3240 reads (reflecting the lowest obtained read number per sample), rarefaction curves indicated a diversity coverage of 85–98% (data not shown). Coinciding with TRFLP, reconstructed total soil microbial community structure was not significantly affected by the ^13^C‐label when comparing labelled and unlabelled samples (data not shown).

**Figure 3:**
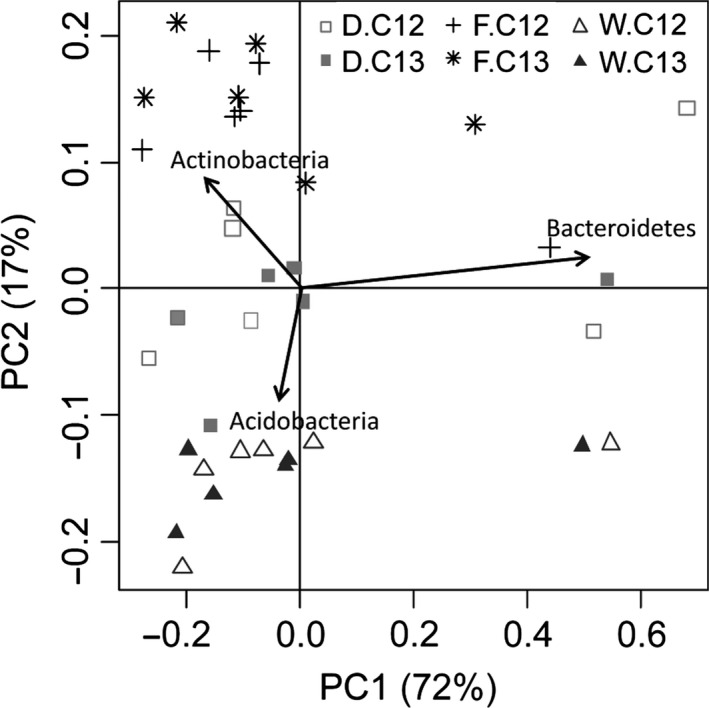
PCA plot generated from partial 16S rRNA gene sequences assigned to phylum level obtained from 6 gradient fractions of DNA directly extracted from soil D, F and W obtained from unlabelled (C12) and labelled (C13) microcosms.

In total, 19 phyla were detected with *Actinobacteria* > *Proteobacteria* ≥ *Bacteroidetes* being dominant in all soils, accounting for 65% (soil W) – 86% (soil D) of all reads (Table S2). *Acidobacteria* were low in soil D and F but two‐ to threefold higher in soil W (16% of all reads). While *Proteobacteria* were found in similar abundance in all soils (16%), *Actinobacteria* were highest in soil F (53%) and *Bacteroidetes* were enriched in soil D (19%). Thus, microbial community composition differed clearly between the soils (Fig. 3, Table S2). *Actinobacteria* were dominated by *Actinomycetales* belonging to *Kineosporia*,* Mycobacter* and *Nocardioides*. However, *Mycobacter* accounted for 1.8–2.2% in all soils, *Kineosporia* (2.6%) and *Nocardioides* (4.7%) were increased two‐ to sixfold in soil F compared with the other soils (Table S2). However, 10–19% of *Actinomycetales* could not be classified on genus level. *Flavobacteriales* belonging to *Flavobacteriaceae* were the most abundant *Bacteroidetes* in all soils. In soil F and W, > 95% of *Flavobacteriaceae* were identified as *Flavobacterium* (5.8% and 8.1% of total reads, respectively); in soil D, only 60% could be grouped into this genus (9.3%), while the others remained unclassified. *Sphingobacteria* composing the genus *Adhaeribacter* made up 2.4% in soil F, accounting for 20% of *Bacteroidetes*, but were found < 0.5% in soil D and W (Table S2).

*Proteobacteria* accounted for 16% of total reads in all soils and were dominated by *Gammaproteobacteria* belonging to the orders *Pseudomonadales* and *Xanthomonadales*. Soil D showed a threefold higher number of *Pseudomonadales* (4.3%) but threefold lower number of *Xanthomonadales* (1.1%) when compared with soil F and W. Around 85–93% of *Pseudomonadales* could be classified as either *Acinetobacter* or *Pseudomonas*. *Xanthomonadales* were dominated by *Steroidobacter* in soil D and F (0.4% and 1.2% of total reads, respectively) and *Thermomonas* and *Steroidobacter* in soil W (0.6% and 0.5% of total reads, respectively; Table S2).

**Figure 4:**
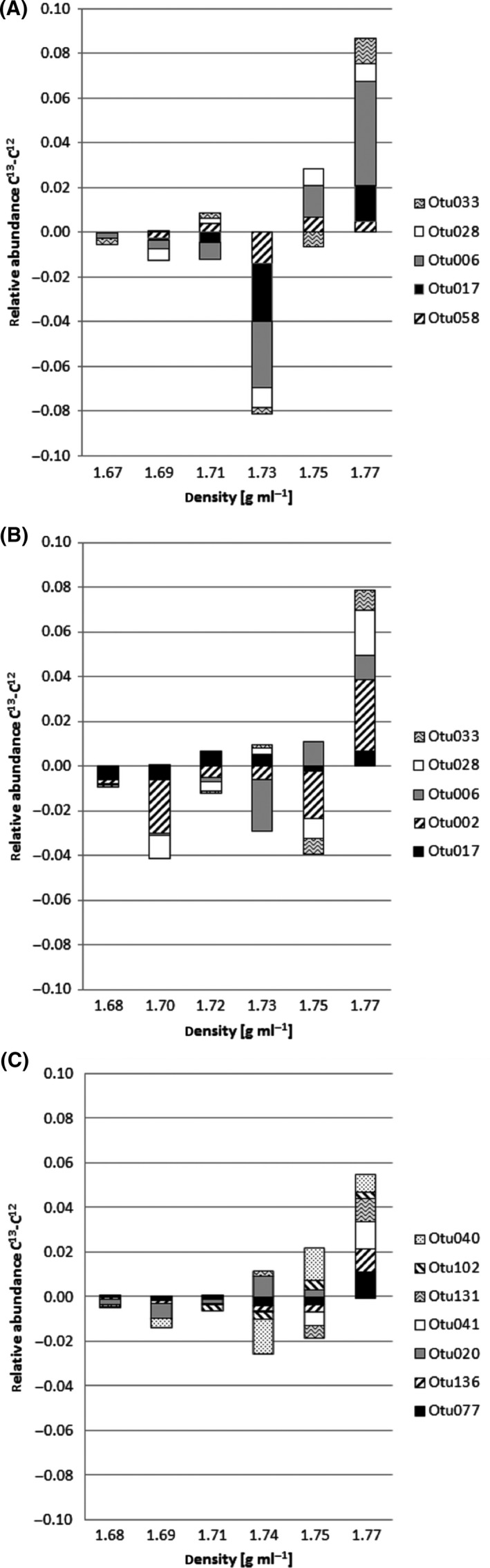
Operational taxonomic units (OTUs) showing shifts towards fractions with higher buoyant density in ^13^C‐labelled microcosms when compared with unlabelled microcosms presented as difference between the relative abundance in ^13^C‐labelled and unlabelled DNA extracts from (A) soil D, (B) soil F and (C) soil W among 6 gradient fractions. Phylogenetic classification of OTUs is given in Table 2.

Screening the diversity pattern of the ^13^C‐labelled soils for shifts in bacterial abundance towards ‘heavier’ fractions when compared with soils spiked with unlabelled ferrocyanide revealed similar density‐resolved shifts in soil D and F while W differed (Fig. 4, Table S2). For both soil D and F, five OTUs were observed in fractions with higher BD in the ^13^C gradient when compared with the unlabelled gradient, from which the following were shifted in both soils: OTU006, OTU017, OTU028 and OTU033, representing bacteria belonging to *Mycobacterium*,* Kineosporia*, unclassified *Actinomycetales* and unclassified *Gammaproteobacteria* respectively (Fig. 4, Table 2). OTU058 (soil D) and OTU002 (soil F) also represented members of *Actinobacteria*, unclassified *Acidimicrobiales* and unclassified *Micromonosporaceae* respectively. For *Actinobacteria*, to which most of the ^13^C‐labelled OTUs belonged to, a phylogenetic tree was constructed showing the distribution of sequences within this phylum (Fig. S3). Diversity pattern of soil W gradients revealed shifts for 7 OTUs representing bacteria classified as *Acidobacteria* (OTU077, Gp3; OTU136, Gp4; OTU0020, Gp6), *Pseudonocardiaceae* (OTU041), *Gemmatimonas* (OTU131) and *Xanthomonadaceae* (OTU102, *Thermomonas*; OTU040, unclassified) (Fig. 4, Table 2).

**Table 2:** List of ^13^C‐labelled OTUs obtained from soil D, F and W and its phylogenetic classification

OTU	Phylum	Class	Oder	Family	Genus
OTU077	Acidobacteria	Acidobacteria_Gp3	incertae_sedis	incertae_sedis	Gp3
OTU136	Acidobacteria	Acidobacteria_Gp4	incertae_sedis	incertae_sedis	Gp4
OTU020	Acidobacteria	Acidobacteria_Gp6	incertae_sedis	incertae_sedis	Gp6
OTU058	Actinobacteria	Actinobacteria	Acidimicrobiales	Unclassified	Unclassified
OTU017	Actinobacteria	Actinobacteria	Actinomycetales	Kineosporiaceae	*Kineosporia*
OTU002	Actinobacteria	Actinobacteria	Actinomycetales	Micromonosporaceae	Unclassified
OTU006	Actinobacteria	Actinobacteria	Actinomycetales	Mycobacteriaceae	*Mycobacterium*
OTU041	Actinobacteria	Actinobacteria	Actinomycetales	Pseudonocardiaceae	Unclassified
OTU028	Actinobacteria	Actinobacteria	Actinomycetales	Unclassified	Unclassified
OTU131	Gemmatimonadetes	Gemmatimonadetes	Gemmatimonadales	Gemmatimonadaceae	*Gemmatimonas*
OTU033	Proteobacteria	Gammaproteobacteria	Unclassified	Unclassified	Unclassified
OTU102	Proteobacteria	Gammaproteobacteria	Xanthomonadales	Xanthomonadaceae	*Thermomonas*
OTU040	Proteobacteria	Gammaproteobacteria	Xanthomonadales	Xanthomonadaceae	Unclassified

## Discussion

In this study, we present the first data on the application of DNA‐SIP to identify microorganisms assimilating C derived from ferrocyanide degradation in soils with different pre‐exposition to iron‐metal complexes applied with deicing salts. Although this method allows the identification of ^13^C‐labelled microbes fulfilling certain ecosystem functions, e.g. biodegradation, by sequencing of nucleic acids directly isolated from soil, it has to be taken into account that it also possesses one main limitation: considerable amounts of the labelled substrate have to be metabolized for a sufficient isotopic enrichment of the nucleic acids, meaning a minimum of two cell divisions in combination with the assimilation of C mainly deriving from the ^13^C substrate (Lueders *et al*., 2004b). This might result in either the need for quite high labelled substrate application not reflecting natural conditions, leading to a biased microbial community, or long incubation times harbouring the risk of cross‐feeding. Therefore, ^13^C‐substrate concentration and incubation time have to be selected with care, best by sampling along a time series as done in the present experiment. Although RNA‐SIP would be a more sensitive approach because RNA synthesis in active cells occurs at high rates and thus ^13^C is incorporated independent of cell replication, it could be shown that isotopically labelled DNA is better resolved via gradient centrifugation than RNA (Lueders *et al*., 2004a). Moreover, DNA‐SIP has the advantage that complete genomes are labelled which allows follow‐up analyses of different functional genes or high‐resolution metagenomics of target groups (Kalyuzhnaya *et al*., 2008).

Although there are various reports of microorganisms capable of degradation of metal‐cyanide complexes using it as nutrient source (Aronstein *et al*., 1994; Barclay *et al*., 1998a,b; Dursun *et al*., 1999; Yanase *et al*., 2000; Kwon *et al*., 2002), several studies neglected controls for photolysis and physical dissociation. It is well established that ferrocyanide complexes are very stable in the dark, with projected half‐lives of 1–1000 years depending on pH, temperature and redox conditions, but decompose rapidly when exposed to daylight with dissociation rates of 8% h^−1^ (Meeussen *et al*., 1992). Upon prolonged UV light exposure, ferrocyanide can undergo sequential photosubstitution reactions to release all cyanide from the iron complex (Kuhn and Young, 2005). As free cyanide (HCN, CN^−^) is adsorbed only weakly or not at all to inorganic soil compounds like sand and clay (Higgins and Dzombak, 2006), it is more bioavailable than ferrocyanide and, consequently, faster degraded (Aronstein *et al*., 1994). Although we cannot absolutely exclude dissociation in our experiment, we suggest that by covering the microcosms with aluminium foil, incubating it in the dark and keeping the water content stable, the liberation of ^13^C‐labelled C sources was mainly due to microbial ferrocyanide degradation. This is in line with a previous study observing a 2.5‐ to 3.5‐fold increase in cyanide reduction when sterile soil slurries were inoculated with different microorganisms (Aronstein *et al*., 1994).

The experiment was conducted with two roadside soils (D, F) and one nearby control soil (W) without anthropogenic pre‐exposition to ferrocyanide complexes in form of deicing salt application in winter. Ferrocyanide application impacted microbial diversity in all soils but with most pronounced effects on soil W. All three soils showed significant differences in microbial community structure 32 days after application of the ferrocyanide, mainly due to the abundance of members of the phyla *Actinobacteria, Bacteroidetes and Acidobacteria*. Nevertheless, our results revealed that despite those differences the bacteria assimilating C derived from ferrocyanide were highly similar among the two roadside soils and belonged mostly to the *Actinomycetales* (*Kineosporia*,* Mycobacterium*, unclassified *Micromonosporaceae* and unclassified *Actinomycetales*). *Actinomycetales* are known as slow growing, typical bulk soil inhabitants playing an important role in the degradation of recalcitrant C (Goodfellow and Williams, 1983; Smalla *et al*., 2001), so it is not surprising that they were efficient degraders of iron‐metal complexes in soil. Coinciding, two facultative autotroph actinomycetes were reported to be capable of growing on cyanide as C and N source previously (Knowles, 1976). Recently, *Actinobacteria* were found to be involved in thiocyanate and cyanide degradation in bioreactors inoculated with sludge from gold ore mining effluent (Kantor *et al*., 2015). However, the observed predominance of ^13^C‐enriched *Actinomycetales* in the roadside soils was surprising as also various *Proteobacteria* are known to have appropriate enzyme systems to degrade cyanide and cyanogenic compounds, e.g. *Pseudomonas* and *Acinetobacter* (Ebbs *et al*., 2006), which were both present in the investigated soils. It can be speculated that *Pseudomonas* and *Acinetobacter*, although possibly involved in ferrocyanide degradation in the present system, used ferrocyanide only as N source and relied on other soil C sources. This is supported by previous findings that *Pseudomonas fluorescens* lacked growth in media containing ferrocyanide as sole C and N source but was able to degrade ferrocyanide when organic nutrients like glucose was added (Dursun *et al*., 1999). Furthermore, *Pseudomonas* sp. are known as highly rhizo‐competent heterotrophs depending on easily degradable C from root exudates (Lugtenberg *et al*., 2001; Berg *et al*., 2005). As the soils used in the present study were collected in November, they probably contained only a low amount of root exudates, resulting in reduced metabolic activity of pseudomonads.

Also in soil W ^13^C‐labelled bacteria were detected, indicating the occurrence of microorganisms in soils capable of ferrocyanide degradation independent of pre‐exposition. However, ^13^C incorporation into microbial biomass C was decreased when compared with the roadside soils, suggesting a slower degradation rate and the need for functional adaptation in soils newly exposed to ferrocyanide. However, it has to be taken into account that ^13^C incorporation into biomass could only serve as a proxy for ferrocyanide degradation but must not reflect the actual removal rate. Moreover, the diversity of ^13^C‐ labelled bacteria differed highly from those of the roadside soils: in soil D and F, ferrocyanide was used as C source mainly by *Actinomycetales*, while in soil W members of *Acidobacteria* (Gp3, Gp4, Gp6), *Gemmatimonadetes* (*Gemmatimonas*) and *Gammaproteobacteria* (*Thermomonas*, unclassified *Xanthomonadaceae*) assimilated the ^13^C. All those taxa were previously found to be enriched in bioreactors containing mining effluent, indicating their capacity to degrade thiocyanate and cyanide (Kantor *et al*., 2015). Surprisingly, neither *Kineosporia* nor *Mycobacterium* assimilated ferrocyanide‐derived C in soil W although their presence at similar abundances when compared with soil D and F. This indicates an adaptation of the microbial community on long‐term ferrocyanide application and consequently its permanent presence as additional C source: in soils first exposed to ferrocyanide, various microorganisms assimilate the new available C but at the end were outcompeted by *Actinomycetales*, resulting in their increased abundance in roadside soils when compared with soil W. However, it has to be taken into account that ferrocyanide is not applied purely to the road environment but as anticaking agent via deicing salts that itself could impact microbial soil community (Cernohlavkova *et al*., 2008; Hofman *et al*., 2012). Although we cannot exclude a long‐term salt related influence, we assume that the impact of previous applied deicers could be neglected in our system as (i) the soil was sampled in November before road deicing salt was first applied in the actual winter period and (ii) salt applied during the last winter was quickly leached due to high precipitation (1280 mm y^−1^) in the sampling area.

Our results indicate that microbial degradation of ferrocyanide complexes could occur in soil without previous photolysis. Moreover, the capacity of ferrocyanide degradation may be present in soil microorganisms independent of anthropogenic pre‐exposition. However, this needs further investigation including (i) abiotic controls to determine the ferrocyanide dissociation rate and (ii) measurement of the actual cyanide removal rate. Although a wide range of bacteria may use ferrocyanide complexes as sole N source, some seem to rely on additional C sources for biodegradation. While in the newly exposed soil bacteria belonging to various phyla assimilate ferrocyanide‐derived C, long‐term exposure favours *Actinomycetales*, indicating, on the one hand, a widespread capacity in soil microorganisms to assimilate cyanogenic compounds and, on the other hand, severe competition for limited resources. Nevertheless, it has to be taken into account that roadside soils probably contain also traffic‐related elements like lead (Pb) which additionally may influence microbial community composition and promote *Actinobacteria* known capable of tolerating heavy metals. In addition, the deicing salt itself could potentially impact microbial diversity pattern, resulting in adaptation to increased Na^+^ in roadside soils. Although we concentrated on the identification of bacteria using ferrocyanide‐derived C in the present study, it is acknowledged that various fungi, e.g. *Fusarium* and *Trichoderma* sp., are capable of metal‐cyanide degradation, too (Barclay *et al*., 1998b). Thus, further analyses should be performed to identify also ^13^C‐labelled fungi to complete the picture of the microbial community assimilating C derived from ferrocyanide. Moreover, also archaea should be taken into account for future studies as they can use methane produced via the reductive pathway of cyanide degradation as C source, thus preventing it from emission to atmosphere and serving as control for greenhouse gas emission. Besides its limitations, DNA‐SIP provides a powerful tool to identify microorganisms capable of a specific function in ecosystems. However, further studies using ^15^N‐labelled and/or ^13^C‐^15^N‐labelled ferrocyanide are necessary to cover all microorganisms using ferrocyanide as nutrient resource. Moreover, RNA‐SIP and metabolic profiling should be included to link microbial ferrocyanide degradation in soil directly to N and C assimilation. Additionally, samples obtained from a time series should be analysed to cover seasonal effects (variation in soil ferrocyanide concentrations during the year, higher amount of easily degradable C in soil during vegetation period, temperature, water content, salinity, etc.).

## Experimental procedures

### Soil description

The experiment was performed using humic topsoil (0–10 cm) of two roadside soils collected in November 2012 at a distance of 10 cm from the tarred road surface at the German federal highway BAB 45 (‘Sauerlandlinie’) in North Rhine‐Westphalia, Germany (50°45′ 56.61″ N/8°09′ 2.8188″ E, named ‘F’, and 50°48′ 21.366″ N/8°05′ 58.6572″ E, named ‘D’). The sites were chosen because (i) BAB 45 has a very high traffic density and (ii) it is 600–700 m a.s.l., resulting in the absolute necessity of deicing salt application due to harsh winter conditions and, consequently, significant ferrocyanide inputs. Soil D consists of 36% sand, 47% silt and 17% clay and is characterized by a pH (CaCl_2_) of 6.1, a water holding capacity (WHC) of 50.2%, total C and N content of 4.7 and 0.3% and a total cyanide content of 2.7 mg kg^−1^. In comparison, soil F consists of 81% sand, 12% silt and 7% clay and is characterized by a pH (CaCl_2_) of 6.8, a WHC of 23.3%, total C and N content of 2.1 and 0.1% and a total cyanide content of 1.5 mg kg^−1^. Additionally, a nearby grassland soil (50°45′ 31.885″ N/8°07′ 47.734″ E, named ‘W’) was collected as control soil without deicing‐salt application, consisting of 19% sand, 58% silt and 23% clay. Soil W has a pH (CaCl_2_) of 5.5, a WHC of 65.3%, total C and N content of 5.1 and 0.5% and a total cyanide content of 0.5 mg kg^−1^. After removal of the herbaceous cover, plant roots and large debris were discarded. The soils were manually homogenized, sieved < 2 mm and stored at 4°C in the dark.

### Microcosm experiment

For each of the soils, 500 g (dry weight) was spiked with 50 mg cyanide using 135.2 mg of the ^13^C‐enriched ferrocyanide K_4_[Fe(^13^CN)_6_] × 3 H_2_O (99 atom% ^13^C, Sigma Aldrich, Hamburg, Germany) and its unlabelled analogue K_4_[Fe(^12^CN)_6_] × 3 H_2_O respectively. To normalize the water content of the soils simultaneously, the ferrocyanide was dissolved in 0.01 M CaCl_2_ in the respective total volume needed to adjust the water content to 60% of WHC. Afterwards, the spiked soils were homogenized for 30 min and filled into 50 ml polyvinylchloride cylinders (30 g dw per cylinder, 9 cylinders per soil and spiking agent) which were sealed at the bottom with a fibrous web (Akkuwik; Pall, Port Washington, New York, USA) and covered with aluminium foil. Control samples without ferrocyanide application were treated similar. The 81 microcosms were placed on silica sand and incubated at 14°C in the dark. To keep the water content between 50% and 60% WHC, microcosms were weighted three times per week and watered when necessary. Sampling occurred after 18, 32 and 74 days in triplicates. Soil subsamples were stored at 4°C for analysis of microbial biomass C and frozen at −80°C for DNA extraction.

### ^13^C incorporation into microbial biomass carbon (C_mic_)

Microbial biomass carbon (C_mic_) was determined by the chloroform‐fumigation method according to Vance *et al*. (1987), using 5 g of soil extracted in 0.01 M CaCl_2_ solution (1:4 w/v) (Joergensen, 1995). Measurement of δ^13^C in the extracts was done via online coupling of liquid chromatography and stable isotope ratio mass spectrometry (LC–IRMS; Thermo Fisher Scientific, Waltham, MA, USA). The δ^13^C values were related to the international V‐PDB standard and computed as follows:

$$\delta {}^{13}C_{\mathrm{mic}}\left(‰\right)=\left[\left(\delta {}^{13}C_{\mathrm{fum}}\times C_{\mathrm{fum}}\right)-\left(\delta {}^{13}C_{\mathrm{nfum}}\times C_{\mathrm{nfum}}\right)\right]/C_{\mathrm{mic}}$$

where C_fum_ and C_nfum_ are C concentrations (in mg L^−1^) of the fumigated and non‐fumigated extracts (Gschwendtner *et al*., 2011). Results were evaluated for statistical significance (*P* < 0.05) using multivariate ANOVA (Adonis function) in R v3.1.2 (http://www.Rproject.org/).

### Nucleic acid extraction, isopycnic centrifugation and fractionation

DNA was extracted from 0.4 g of soil using the protocol described by Lueders *et al*. (2004a) and the Precellys24 Instrument (PeqLab, Erlangen, Germany). Quality and quantity of the nucleic acids were checked on a spectrophotometer (Nanodrop; PeqLab) and gel electrophoresis. Isopycnic centrifugation was performed in CsCl gradients containing 5 ml of CsCl stock solution (1.84 g ml^−1^) and 1 ml of gradient buffer (0.1 M Tris‐HCl, pH 8; 0.1 M KCl; 1 mM EDTA) including 4 μg of DNA at 20°C at 45 000 r.p.m. for 48 h in a VTI 65.2 vertical rotor (Beckman Coulter, Krefeld, Germany). Prior to centrifugation, density of the gradients was checked via AR200 digital refractometer (Reichert Technologies, Munich, Germany) and adjusted to 1.72 g ml^−1^ (Lueders *et al*., 2004a). Centrifuged gradients were fractionated from bottom to top into 12 equal fractions using Perfusor compact S (Braun AG, Melsungen, Germany). The density of each fraction was measured with a refractometer. Afterwards, the fractions were purified as described by Lueders *et al*. (2004a) and nucleic acids were quantified using a PicoGreen assay.

### Quantification of 16S rRNA genes

Bacterial 16S rRNA genes in gradient fractions were determined by quantitative real‐time PCR (qPCR) in an ABI 7300 Cycler (Life Technologies, Darmstadt, Germany) using the primers FP16S/RP16S (Bach *et al*., 2002). Each 25 μl of PCR reaction contained 1× of Power SYBR Green PCR master mix (Life Technologies), 0.2 μM of each primer (Metabion, Martinsried, Germany), 15 μg of BSA (Sigma Aldrich, Taufkirchen, Germany) and 2 μl of template DNA. Thermal cycling started with an initial denaturation step at 95°C for 10 min, followed by 40 cycles of amplification (95°C for 45 s, 58°C for 45 s, 72°C for 45 s) and a melting curve analysis to confirm the specificity of the SYBR Green‐quantified amplicons. Serial dilutions (10^1^ to 10^6^ copies μl^−1^) of plasmid DNA containing the PCR product of the 16 rRNA gene of *Pseudomonas putida* were used to calculate standard curves. The amplification efficiency, calculated with the formula Eff = 10^(−1/slope)^ − 1, was 94%.

### TRFLP analysis

For the amplification of 1.4 kb fragments of the 16S rRNA gene the primers 27f (FAM‐labelled) and 1401r (Gschwendtner *et al*., 2015) were used with the following PCR conditions: 94°C for 10 min for initial denaturation, followed by 30 cycles of amplification (94°C for 1 min, 58°C for 1 min, 72°C for 1.5 min) and a final extension step at 72°C for 10 min. PCR reactions (total volume 50 μl) contained 1× of Taq buffer (Life Technologies), 2.5 mM of MgCl_2_ (Fermentas, St. Leon Rot, Germany), 0.2 mM of dNTPs (Fermentas), 0.2 μM of each primer (Metabion), 150 μg of BSA (Sigma Aldrich), 2.5 U of Taq Polymerase (Life Technologies) and 20 ng of template DNA. Amplicons were purified using Nucleospin Gel and PCR Cleanup kit (Macherey Nagel, Düren, Germany), digested with MspI (Fermentas) and separated on an ABI 3730 sequencer using MapMarker 1000 (Eurogentec, Köln, Germany) as internal standard (Gschwendtner *et al*., 2015). TRFLP profiles were analysed in r v3.1.2 (http://www.Rproject.org/) using multivariate ANOVA (Adonis function) by calculating the relative abundance of TRFs normalized by the total signal height of the respective TRF patterns. Fragments smaller than 50 bases and TRFs contributing <1% to the total peak height were excluded from the analysis.

### Sequencing analysis

Sample selection for 454 pyrosequencing analysis was done based on TRFLP results as it could be shown that TRFLP and 454 pyrosequencing are capable of recovering highly comparable community structure (Pilloni *et al*., 2012). Therefore, amplicon sequencing of every second gradient fraction (1, 3, 5, 7, 9, 11) for one randomly selected sample each soil was performed on a 454 GS FLX Titanium system (Roche, Penzberg, Germany) using the universal eubacterial primers 27f and 984r (5′‐gta agg ttc ytc gcg‐3′, reverse complement of modified primer Ba967Fd, Klindworth *et al*., 2013) extended with unique Multiplex Identifiers, a four base library key and adapters for sample identification. Each 25 μl of PCR reaction was done in triplicates and contained 1× of PCR buffer with 1.8 mM of MgCl_2_, 2 mM of dNTPs, 1.25 U of high fidelity polymerase (Roche, Penzberg, Germany), 0.2 μM of each primer (Metabion), 60 μg of BSA (Sigma Aldrich) and 5 ng of the purified PCR products from previous TRFLP as template DNA. PCR conditions were initial denaturation (95°C, 5 min), followed by 10 cycles of denaturation (94°C, 1 min), annealing (52°C, 1 min) and elongation (72°C, 1 min), ending with a final extension (72°C, 10 min). Triplicates were pooled after PCR, purified using Gel and PCR Cleanup Kit (Macherey Nagel) and quantified via PicoGreen assay. After measuring fragment size and concentration via Bioanalyzer 2100 device on a DNA 7500 chip (Agilent, Böblingen, Germany), samples were pooled in an equimolar ratio of 10^9^ molecules μl^−1^. Subsequently, an emulsion PCR was performed (Gschwendtner *et al*., 2015). Sequence data were processed in mothur v.1.33.3 (Schloss, 2009) using the same dataset as reference for chimera check and the Ribosomal Database Project dataset for phylogenetic classification. The calculated distance matrix resulted in OTUs obtained by the nearest neighbour clustering algorithm at 97% sequence similarity (Gschwendtner *et al*., 2015) and was used for calculating rarefaction curves. For analysis of *Actinobacteria* (to which most of the bacteria assimilating ferrocyanide‐derived C in soils D and F belonged to), representative sequences of each OTU > 0.5% of all *Actinobacteria* were aligned to the silva database for construction of a phylogenetic tree in ARB (Ludwig *et al*., 2004). The nucleotide sequence data obtained in this study have been submitted to the GenBank database under accession numbers SRR2469384, SRR2469388, SRR2469393, SRR2469395, SRR2469397, SRR2469405, SRR2469407, SRR2469409, SRR2469412, SRR2469450, SRR2469452, SRR2469453, SRR2469456, SRR2469457, SRR2469458, SRR2469460, SRR2469461, SRR2469462, SRR2469463, SRR2469464, SRR2469465, SRR2469467, SRR2469468, SRR2469470, SRR2469471, SRR2469473, SRR2469476, SRR2469478, SRR2469479, SRR2469482, SRR2469484, SRR2469486, SRR2469487, SRR2469488, SRR2469489, SRR2469490.

## Supporting information


**Fig. S1.** Abundance of 16S rRNA genes among 12 gradient fractions of DNA extracted from (A) soil D, (B) soil F and (C) soil W after 18, 32 and 74 days of ^13^C‐labelled and unlabelled ferrocyanide application quantified via real‐time PCR.

**Fig. S2.** UPGMA dendrogram generated from TRFLP profiles based on 16S rRNA gene amplicons from 12 gradient fractions (numbered 1–12) of DNA extracted from (A) soil D, (B) soil F and (C) soil W obtained from unlabelled (C12) and labelled (C13) microcosms (*n* = 3). Buoyant density of the gradient fractions increased with decreasing fraction number. The scale indicates the distance level.

**Fig. S3.** Phylogenetic dendrogram (maximum likelihood consensus tree) showing the distribution of sequences related to *Actinobacteria* derived from soil D, F and W at day 32 after ferrocyanide application. OTUs representing bacteria assimilating ferrocyanide‐derived C are highlighted in grey.

**Table S1.** Relative abundance (%) of TRFLP fragments obtained from the reconstructed TRFLP profile by summarized gradient fractions from soil D, F and W based on partial 16S rRNA gene sequences after DNA extraction and PCR amplification. TRFs which are ^13^C‐labelled are highlighted in grey.

**Table S2.** Relative abundance (%) of sequences > 1% in at least one gradient fraction assigned to genus level obtained from the reconstructed OTU profile by summarized gradient fractions from soil D, F and W based on partial 16S rRNA gene sequences after DNA extraction and PCR amplification. Genera containing ^13^C‐labelled OTUs are highlighted in grey.

**Table S3.** Relative abundance (%) of sequences > 1% in at least one gradient fraction assigned to genus level obtained from 6 gradient fractions of DNA extracted from 13C‐labelled and unlabelled soil D, F and W based on partial 16S rRNA gene sequences after PCR amplification. Buoyant density increased among fractions 11 < 9 < 7 < 5 < 3 < 1. Genera containing ^13^C‐labelled OTUs are highlighted in grey.

## References

[mbt212362-bib-0001] Alesii, B.A. , and Fuller, W.H. (1976) The mobility of three cyanide forms in soil Proceedings of the Residual Management by Land Disposal, Hazardous Waste Research Symposium. Fuller W.H. (ed). Cincinatti: U.S. Environmental Protection Agency, pp. 213–223.

[mbt212362-bib-0002] Allen, J. , and Strobel, G.A. (1966) The assimilation of H^14^CN by a variety of fungi. Can J Microbiol 12: 414–416. 5929162 10.1139/m66-056

[mbt212362-bib-0003] Aronstein, B.N. , Maka, A. , and Srivastava, V.J. (1994) Chemical and biological removal of cyanides from aqueous and soil‐containing systems. Appl Microbiol Biotechnol 41: 700–707.

[mbt212362-bib-0004] Bach, H.J. , Tomanova, J. , Schloter, M. , and Munch, J.C. (2002) Enumeration of total bacteria and bacteria with genes for proteolytic activity in pure cultures and in environmental samples by quantitative PCR mediated amplification. J Microbiol Methods 49: 235–245. 11869788 10.1016/s0167-7012(01)00370-0

[mbt212362-bib-0005] Barclay, M. , Tett, V.A. , and Knowles, C.J. (1998a) Metabolism and enzymology of cyanide/metallocyanide biodegradation by *Fusarium solani* under neutral and acidic conditions. Enzyme Microb Technol 23: 321–330.

[mbt212362-bib-0006] Barclay, M. , Hart, A. , Knowles, C.J. , Meeussen, J.C.L. , and Tett, V.A. (1998b) Biodegradation of metal cyanides by mixed and pure cultures of fungi. Enzyme Microb Technol 22: 223–231.

[mbt212362-bib-0007] Berg, G. , Eberl, L. , and Hartmann, A. (2005) The rhizosphere as a reservoir for opportunistic human pathogenic bacteria. Environ Microbiol 7: 1673–1685. 16232283 10.1111/j.1462-2920.2005.00891.x

[mbt212362-bib-0008] Berthold, D.A. , Andersson, M.E. , and Nordlund, P. (2000) New insight into the structure and function of the alternative oxidase. Biochim Biophys Acta 1460: 241–254. 11106766 10.1016/s0005-2728(00)00149-3

[mbt212362-bib-0009] Cernohlavkova, J. , Hofman, J. , Bartos, T. , Sanka, M. , and Andel, P. (2008) Effects of road deicing salts on soil microorganisms. Plant Soil Environ 54: 479–485.

[mbt212362-bib-0010] Dubey, S.K. , and Holmes, D.S. (1995) Biological cyanide destruction mediated by microorganisms. World J Microbiol Biotechnol 11: 257–265. 24414644 10.1007/BF00367095

[mbt212362-bib-0011] Dursun, A.Y. , Calik, A. , and Aksu, Z. (1999) Degradation of ferrous(II) cyanide complex ions by *Pseudomonas fluorescens* . Process Biochem 34: 901–908.

[mbt212362-bib-0012] Ebbs, S.D. , Wong‐Chong, G.M. , Bond, B.S. , Bushey, J.T. , and Neuhauser, E.F. (2006) Biological transformation of cyanide in water and soil In Cyanide in Water and Soil. Dzombak D.A., Ghosh R.S., and Wong‐Chong G.M. (eds). Boca Raton: CRC Press, pp. 93–121.

[mbt212362-bib-0013] Gallagher, L.A. , and Manoil, C. (2001) *Pseudomonas aeruginosa* PAO1 kills Caenorhabditis elegans by cyanide poisoning. J Bacteriol 183: 6207–6214. 11591663 10.1128/JB.183.21.6207-6214.2001 PMC100099

[mbt212362-bib-0014] Goodfellow, M. , and Williams, S.T. (1983) Ecology of actinomycetes. Annu Rev Microbiol 37: 189–216. 6357051 10.1146/annurev.mi.37.100183.001201

[mbt212362-bib-0015] Gschwendtner, S. , Esperschütz, J. , Buegger, F. , Reichmann, M. , Müller, M. , Munch, J.C. , and Schloter, M. (2011) Effects of genetically modified starch metabolism in potato plants on photosynthate fluxes into the rhizosphere and on microbial degraders of root exudates. FEMS Microbiol Ecol 76: 564–575. 21348886 10.1111/j.1574-6941.2011.01073.x

[mbt212362-bib-0016] Gschwendtner, S. , Leberecht, M. , Engel, M. , Kublik, S. , Dannenmann, M. , Polle, A. , and Schloter, M. (2015) Effects of elevated atmospheric CO_2_ on microbial community structure at the plant‐soil interface of young beech trees (*Fagus sylvatica* L.) grown at two sites with contrasting climatic conditions. Microb Ecol 69: 867–878. 25370887 10.1007/s00248-014-0527-x

[mbt212362-bib-0017] Higgins, C.J. , and Dzombak, D.A. (2006) Free cyanide sorption on freshwater sediment and model components. Soil Sediment Contam 15: 497–510.

[mbt212362-bib-0018] Hofman, J. , Travnickova, E. , and Andel, P. (2012) Road salts effects on soil chemical and microbial properties at grassland and forest site in protected natural areas. Plant Soil Environ 58: 282–288.

[mbt212362-bib-0019] Joergensen, R.G. (1995) The fumigation‐extraction method to estimate soil microbial biomass: extraction with 0.01M CaCl_2_ . Agribiol Res 48: 319–324.

[mbt212362-bib-0020] Jünemann, S. (1997) Cytochrome bd terminal oxidase. Biochim Biophys Acta 1321: 107–127. 9332500 10.1016/s0005-2728(97)00046-7

[mbt212362-bib-0021] Kalyuzhnaya, M.G. , Lapidus, A. , Ivanova, N. , Copeland, A.C. , McHardy, A.C. , Szeto, E. , *et al* (2008) High‐resolution metagenomics targets specific functional types in complex microbial communities. Nat Biotechnol 26: 1029–1034. 18711340 10.1038/nbt.1488

[mbt212362-bib-0022] Kantor, R.S. , van Zyl, A.W. , van Hille, R.P. , Thomas, B.C. , Harrison, S.T.L. , and Banfield, J.F. (2015) Bioreactor microbial ecosystems for thiocyanate and cyanide degradation unravelled with genome‐resolved metagenomics. Environ Microbiol 17: 4929–4941. 26031303 10.1111/1462-2920.12936

[mbt212362-bib-0023] Klindworth, A. , Pruesse, E. , Schweer, T. , Peplies, J. , Quast, C. , Horn, M. , and Glöckner, F.O. (2013) Evaluation of general 16S ribosomal RNA gene PCR primers for classical and next‐generation sequencing‐based diversity studies. Nucl Acids Res 41: e1–e1. 22933715 10.1093/nar/gks808 PMC3592464

[mbt212362-bib-0024] Knowles, C.J. (1976) Microorganisms and cyanide. Bacteriol Rev 40: 652–680. 791236 10.1128/br.40.3.652-680.1976 PMC413975

[mbt212362-bib-0025] Korte, F. , Spiteller, M. , and Coulston, F. (2000) The cyanide leaching gold recovery process is a nonsustainable technology with unacceptable impacts on ecosystems and humans: the disaster in Romania. Ecotoxicol Environ Saf 46: 241–245. 10903819 10.1006/eesa.2000.1938

[mbt212362-bib-0026] Kuhn, D.D. , and Young, T.C. (2005) Photolytic degradation of hexacyanoferrate (II) in aqueous media: the determination of the degradation kinetics. Chemosphere 60: 1222–1230. 16018892 10.1016/j.chemosphere.2005.02.011

[mbt212362-bib-0027] Kwon, H.K. , Woo, S.H. , and Park, J.M. (2002) Degradation of tetracyanonickelate (II) by *Cryptococcus humicolus* MCN2. FEMS Microbiol Lett 214: 211–216. 12351233 10.1111/j.1574-6968.2002.tb11349.x

[mbt212362-bib-0028] Ludwig, W. , Strunk, O. , Westram, R. , Richter, L. , Meier, H. , Yadhukumar , *et al* (2004) ARB: a software environment for sequence data. Nucl Acids Res 32: 1363–1371. 14985472 10.1093/nar/gkh293 PMC390282

[mbt212362-bib-0029] Lueders, T. , Manefield, M. , and Friedrich, M.W. (2004a) Enhanced sensitivity of DNA‐ and rRNA‐based stable isotope probing by fractionation and quantitative analysis of isopycnic centrifugation gradients. Environ Microbiol 6: 73–78. 14686943 10.1046/j.1462-2920.2003.00536.x

[mbt212362-bib-0030] Lueders, T. , Wagner, B. , Claus, P. , and Friedrich, M.W. (2004b) Stable isotope probing of rRNA and DNA reveals a dynamic methylotroph community and trophic interactions with fungi and protozoa in oxic rice field soil. Environ Microbiol 6: 60–72. 14686942 10.1046/j.1462-2920.2003.00535.x

[mbt212362-bib-0031] Lugtenberg, B.J.J. , Dekkers, L. , and Bloemberg, G.V. (2001) Molecular determinants of rhizosphere colonization by *Pseudomonas* . Annu Rev Phytopathol 39: 461–490. 11701873 10.1146/annurev.phyto.39.1.461

[mbt212362-bib-0032] Mansfeldt, T. (2001) Cyanide in paper de‐inking sludge used as a soil amendment. J Plant Nutr Soil Sci 164: 637–641.

[mbt212362-bib-0033] Mansfeldt, T. , and Dohrmann, R. (2001) Identification of a crystalline cyanide‐containing compound in blast furnace sludge deposits. J Environ Qual 30: 1927–1932. 11789998 10.2134/jeq2001.1927

[mbt212362-bib-0034] Mansfeldt, T. , Gehrt, S.B. , and Friedl, J. (1998) Cyanides in a soil of a former coking plant site. J Plant Nutr Soil Sci 161: 229–234.

[mbt212362-bib-0035] Meeussen, J.C.L. , Keizer, M.G. , and De Haan, F.A.M. (1992) Chemical stability and decomposition rate of iron cyanide complexes in soil solutions. Environ Sci Technol 26: 511–516.

[mbt212362-bib-0036] Møller, B.L. , and Seigler, D.S. (1998) Biosynthesis of cyanogenic glucosides, cyanolipids, and related compounds In Plant Amino Acids: Biochemistry and Biotechnology. Singh B.K. (ed). New York: Marcel Dekker, pp. 593–609.

[mbt212362-bib-0037] Ohno, T. (1990) Levels of total cyanide and NaCl in surface waters adjacent to road salt storage facilities. Environ Poll 67: 123–132. 10.1016/0269-7491(90)90077-p 15092217

[mbt212362-bib-0038] Paschka, M.G. , Ghosh, R.S. , and Dzombak, D.A. (1999) Potential water‐quality effects from iron cyanide anticaking agents in road salt. Water Environ Res 71: 1235–1239.

[mbt212362-bib-0039] Pilloni, G. , Granitsiotis, M.S. , Engel, M. , and Lueders, T. (2012) Testing the limits of 454 pyrotag sequencing: reproducibility, quantitative assessment and comparison to T‐RFLP fingerprinting of aquifer microbes. PLoS ONE 7: e40467. 22808168 10.1371/journal.pone.0040467 PMC3395703

[mbt212362-bib-0040] Raybuck, S.A. (1992) Microbes and microbial enzymes for cyanide degradation. Biodegrad 3: 3–18. 10.1007/BF00189632 1369135

[mbt212362-bib-0041] Rennert, T. , Kaufhold, S. , and Mansfeldt, T. (2007) Identification of iron‐cyanide complexes in contaminated soils and wastes by Fourier transform infrared spectroscopy. Environ Sci Technol 41: 5266–5270. 17822089 10.1021/es070492g

[mbt212362-bib-0042] Richardson, D.J. (2000) Bacterial respiration: a flexible process for a changing environment. Microbiol 146: 551–571. 10.1099/00221287-146-3-551 10746759

[mbt212362-bib-0043] Schloss, P.D. (2009) A high‐throughput DNA sequence aligner for microbial ecology studies. PLoS ONE 4: e8230 20011594 10.1371/journal.pone.0008230 PMC2788221

[mbt212362-bib-0044] Smalla, K. , Wieland, G. , Buchner, A. , Zock, A. , Parzy, J. , Kaiser, S. , *et al* (2001) Bulk and rhizosphere soil bacterial communities studied by denaturing gradient gel electrophoresis: plant‐dependent enrichment and seasonal shifts revealed. Appl Environ Microbiol 67: 4742–4751. 11571180 10.1128/AEM.67.10.4742-4751.2001 PMC93227

[mbt212362-bib-0045] Solomonson, L.P. (1981) Cyanide as a metabolic inhibitor In Cyanide in Biology. Vennesland B., Conn E.E., Knowles C.J., Westley J., and Wissing F. (eds). New York: Academic Press, pp. 11–28.

[mbt212362-bib-0046] Sut, M. , Fischer, T. , Repmann, F. , and Raab, T. (2013) Long‐term release of iron‐cyanide complexes from the soils of a manufactured gas plant site. J Environ Protect 4: 8–19. 10.1080/10934529.2015.981116 25594121

[mbt212362-bib-0047] Thatcher, R.C. , and Weaver, T.L. (1976) C‐N cycling through microbial formamide metabolism. Science (New York, NY) 192: 1234–1235. 10.1126/science.192.4245.1234 17771758

[mbt212362-bib-0048] Vance, E.D. , Brooks, P.C. , and Jenkinson, D.S. (1987) An extraction method for measuring soil microbial biomass C. Soil Biol Biochem 19: 703–707.

[mbt212362-bib-0049] Wehrer, M. , Rennert, T. , Mansfeldt, T. , and Totsche, K.U. (2011) Contaminants at former manufactured gas plants: sources, properties, and processes. Crit Rev Environ Sci Technol 41: 1883–1969.

[mbt212362-bib-0050] Wong‐Chong, G.M. , Nakles, D.V. , and Luthy, R.G. (2006) Manufacture and the use of cyanide In Cyanides in Water and Soil. Dzombak D.A., Ghosh R.S., and Wong‐Chong G.M. (eds). Boca Raton, FL: CRC Press, pp. 41–54.

[mbt212362-bib-0051] Yanase, H. , Sakamoto, A. , Okamoto, K. , Kita, K. , and Sato, Y. (2000) Degradation of the metal‐cyano complex tetracyanonickelate (II) by *Fusarium oxysporum* N‐10. Appl Microbiol Biotechnol 53: 328–334. 10772474 10.1007/s002530050029

[mbt212362-bib-0052] Zagrobelny, M. , Bak, S. , and Moller, B.L. (2008) Cyanogenesis in plants and arthropods. Phytochemistry 69: 1457–1468. 18353406 10.1016/j.phytochem.2008.02.019

