# Supplementary figures and images for: Long‐term ferrocyanide application via deicing salts promotes the establishment of Actinomycetales assimilating ferrocyanide‐derived carbon in soil
Source: Microb Biotechnol. 2016 May 19;9(4):502–13. doi: 10.1111/1751-7915.12362 (PMC4919992; doi:10.1111/1751-7915.12362)

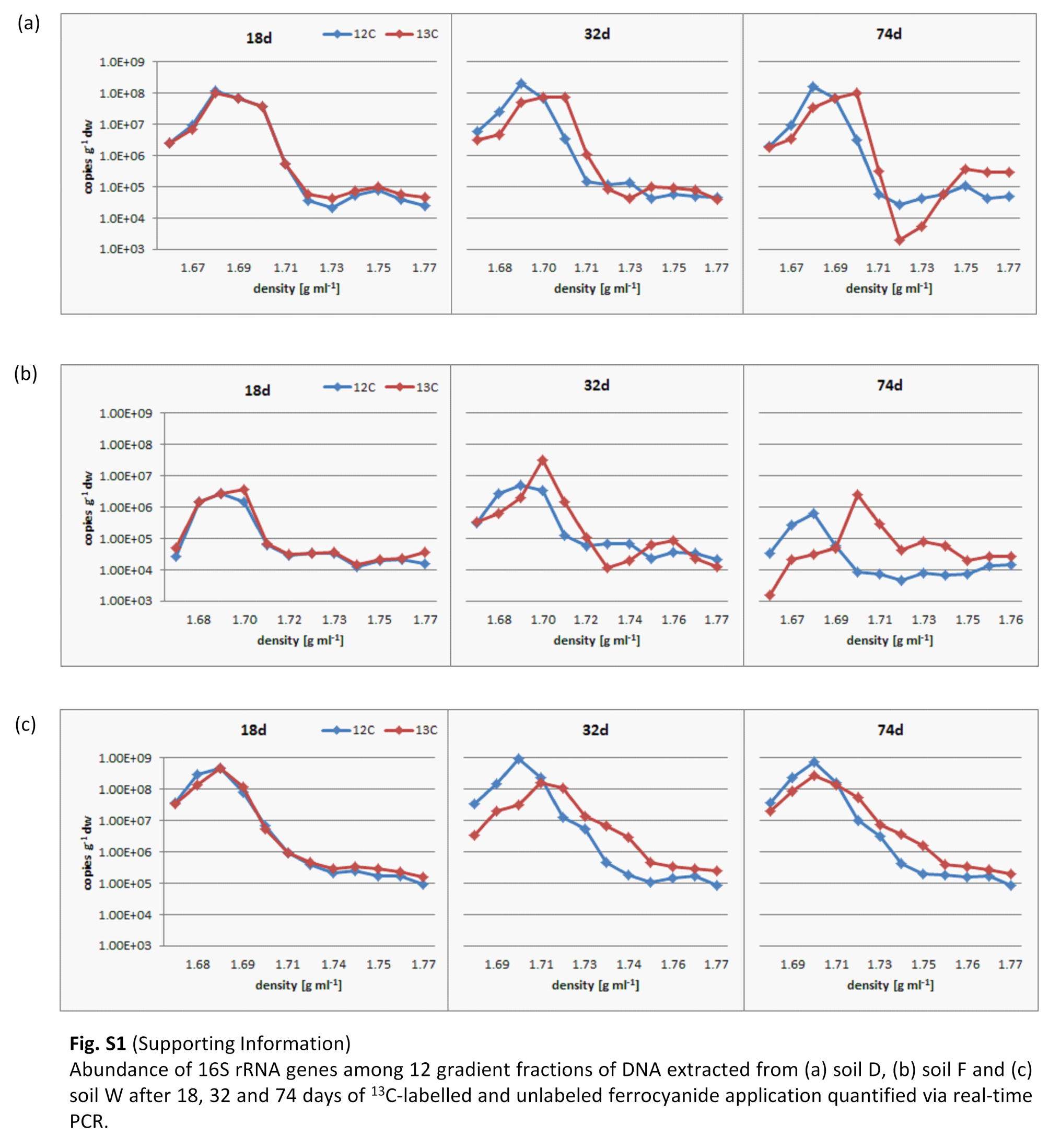

Supplement: Supplementary file 1 — Fig. S1. Abundance of 16S rRNA genes among 12 gradient fractions of DNA extracted from (A) soil D, (B) soil F and (C) soil W after 18, 32 and 74 days of 13C‐labelled and unlabelled ferrocyanide application quantified via real‐time PCR. [file MBT2-9-502-s001.tif]

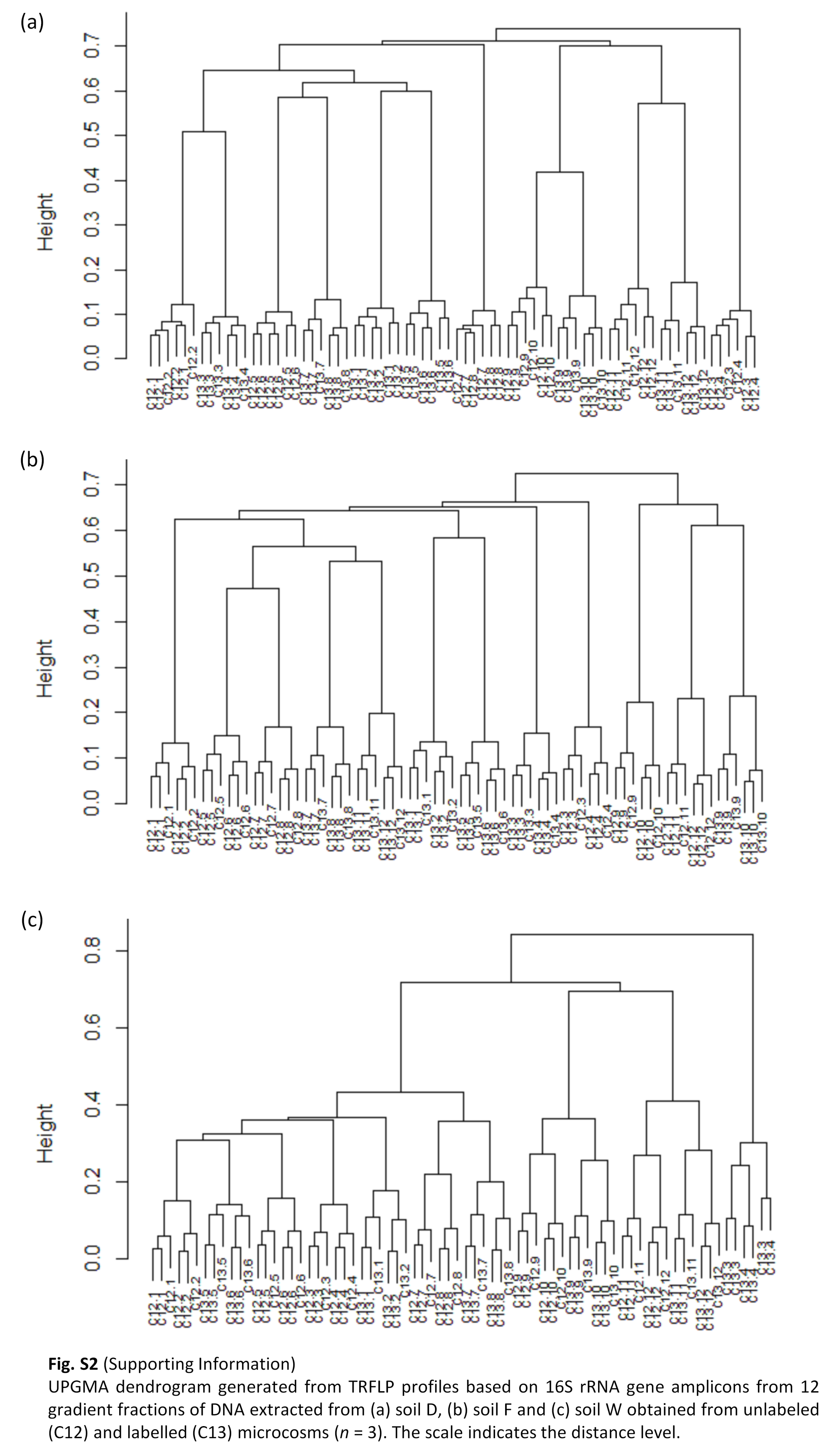

Supplement: Supplementary file 2 — Fig. S2. UPGMA dendrogram generated from TRFLP profiles based on 16S rRNA gene amplicons from 12 gradient fractions (numbered 1–12) of DNA extracted from (A) soil D, (B) soil F and (C) soil W obtained from unlabelled (C12) and labelled (C13) microcosms (n = 3). Buoyant density of the gradient fractions increased with decreasing fraction number. The scale indicates the distance level. [file MBT2-9-502-s002.tif]
