# Supplementary material for: Long‐term ferrocyanide application via deicing salts promotes the establishment of Actinomycetales assimilating ferrocyanide‐derived carbon in soil
Source: Microb Biotechnol. 2016 May 19;9(4):502–13. doi: 10.1111/1751-7915.12362 (PMC4919992; doi:10.1111/1751-7915.12362)

Fig. S3 (Supporting Information) Phylogenetic dendrogram (maximum likelihood consensus tree) showing the distribution of sequences related to Actinobacteria derived from soil D, F and W at day 32 after ferrocyanide application. OTUs representing bacteria assimilating ferrocyanide-derived C are highlighted in grey.

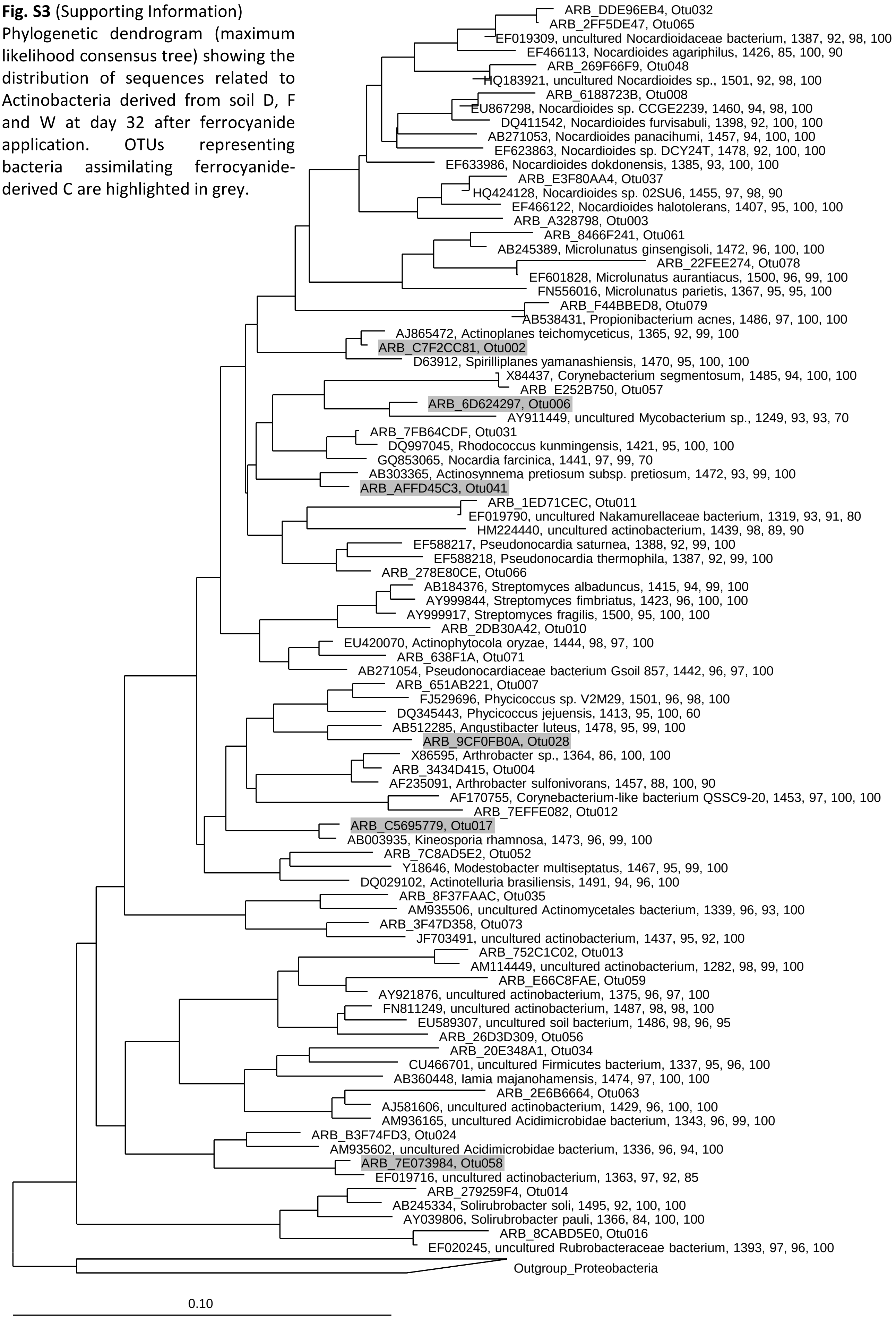

Supplement: Supplementary file 3 — Fig. S3. Phylogenetic dendrogram (maximum likelihood consensus tree) showing the distribution of sequences related to Actinobacteria derived from soil D, F and W at day 32 after ferrocyanide application. OTUs representing bacteria assimilating ferrocyanide‐derived C are highlighted in grey. [file MBT2-9-502-s003.pdf]
