# Supplementary material for: Long‐term ferrocyanide application via deicing salts promotes the establishment of Actinomycetales assimilating ferrocyanide‐derived carbon in soil
Source: Microb Biotechnol. 2016 May 19;9(4):502–13. doi: 10.1111/1751-7915.12362 (PMC4919992; doi:10.1111/1751-7915.12362)
Supplement: Supplementary file 4 — Table S1. Relative abundance (%) of TRFLP fragments obtained from the reconstructed TRFLP profile by summarized gradient fractions from soil D, F and W based on partial 16S rRNA gene sequences after DNA extraction and PCR amplification. TRFs which are 13C‐labelled are highlighted in grey. [file MBT2-9-502-s004.pdf]

Table S1

Relative abundance (%) of TRFLP fragments obtained from the reconstructed TRFLP profile by summarized gradient fractions from soil D, F and W based on partial 16S rRNA gene sequences after DNA extraction and PCR amplification. TRFs which are ^{13}C-labelled are highlighted in grey.

TRF	D	F	W
B53	1.65	2.58	0.37
B56	39.06	33.36	19.07
B60	2.13	2.60	1.42
B64	1.59	3.10	1.40
B69	1.80	3.37	1.05
B73	1.58	2.60	0.88
B77	2.56	2.54	0.98
B82	3.49	2.85	2.16
B86	1.07	0.95	0.63
B89	0.83	0.91	0.50
B93	1.56	0.95	0.42
B99	2.17	2.22	0.45
B103	0.73	1.12	0.27
B107	0.24	0.36	0.22
B111	0.32	1.71	0.26
B114	0.81	1.20	0.23
B118	1.31	1.03	1.53
B122	1.82	2.40	47.55
B127	3.22	2.20	2.22
B131	0.30	0.59	0.14
B136	1.18	2.04	0.92
B139	2.39	1.58	1.97
B144	0.65	1.99	0.38
B148	5.63	3.48	2.19
B151	0.45	0.76	0.00
B158	1.70	1.44	0.87
B161	0.95	1.75	0.40
B164	0.23	0.21	0.02
B168	0.79	0.96	0.83
B171	0.37	0.27	0.20
B175	0.37	0.17	0.00
B182	0.21	0.34	0.02
B187	0.33	0.41	0.04
B190	0.18	0.78	0.40
B196	0.27	0.19	0.29
B199	0.75	0.13	0.30
B204	0.73	0.25	0.20
B207	0.46	0.17	0.10
B211	0.48	0.54	0.07
B214	0.29	0.00	0.00
B219	0.09	0.03	0.12
B224	0.80	0.84	0.26
B227	0.35	0.00	0.10
B230	0.34	0.65	0.02
B234	0.00	0.06	0.00

B236	0.04	0.09	0.00
B241	0.10	0.19	0.05
B244	0.16	0.00	0.00
B249	0.00	0.47	0.11
B258	0.85	0.00	0.00
B263	0.38	0.27	0.52
B267	0.10	0.11	0.00
B275	0.32	0.71	0.19
B279	1.01	1.32	0.36
B282	0.00	0.09	0.22
B287	0.56	0.23	0.50
B290	0.00	0.00	0.16
B293	0.18	0.30	0.41
B301	0.00	0.17	0.00
B304	0.13	0.06	0.00
B322	0.10	0.53	0.03
B331	0.62	0.00	0.19
B338	0.06	0.00	0.00
B370	0.00	0.07	0.00
B377	0.10	0.00	0.00
B380	0.01	0.00	0.16
B384	0.00	0.09	0.00
B398	0.04	0.30	0.00
B401	0.12	0.70	0.00
B422	0.10	0.04	0.14
B427	0.06	0.08	0.25
B433	0.32	0.08	0.33
B438	0.78	0.77	0.27
B446	0.11	0.09	0.34
B455	0.09	0.07	0.24
B462	0.04	0.04	0.16
B468	0.06	0.61	0.13
B472	0.11	0.11	0.21
B480	0.00	0.00	0.09
B488	0.95	0.81	0.40
B492	0.63	0.59	0.05
B495	0.17	0.34	0.14
B500	0.14	0.15	0.19
B505	0.30	0.15	0.43
B509	0.17	0.23	0.21
B516	0.54	0.89	0.48
B520	0.04	0.04	0.06
B526	0.26	0.30	0.47
B533	0.37	0.18	0.17
B538	0.22	0.15	0.07
B561	0.04	0.06	0.19
B576	0.06	0.08	0.05
B579	0.00	0.00	0.06
B586	0.00	0.05	0.00
B592	0.03	0.06	0.00
B645	0.00	0.06	0.00
B802	0.21	0.00	0.15
