# Supplementary material for: Long‐term ferrocyanide application via deicing salts promotes the establishment of Actinomycetales assimilating ferrocyanide‐derived carbon in soil
Source: Microb Biotechnol. 2016 May 19;9(4):502–13. doi: 10.1111/1751-7915.12362 (PMC4919992; doi:10.1111/1751-7915.12362)
Supplement: Supplementary file 5 — Table S2. Relative abundance (%) of sequences > 1% in at least one gradient fraction assigned to genus level obtained from the reconstructed OTU profile by summarized gradient fractions from soil D, F and W based on partial 16S rRNA gene sequences after DNA extraction and PCR amplification. Genera containing 13C‐labelled OTUs are highlighted in grey. [file MBT2-9-502-s005.pdf]

Table S2

Relative abundance (%) of sequences >1% in at least one gradient fraction assigned to genus level obtained from the reconstructed OTU profile by summarized gradient fractions from soil D, F and W based on partial 16S rRNA gene sequences after DNA extraction and PCR amplification. Genera containing ^{13}C-labelled OTUs are highlighted in grey.

phylum	class	order	family	genus	D	F	W
Acidobacteria	Acidobacteria_Gp1	incertae_sedis	incertae_sedis	*Gp1*	1.07	0.02	1.01
Acidobacteria	Acidobacteria_Gp3	incertae_sedis	incertae_sedis	*Gp3*	1.70	1.39	3.00
Acidobacteria	Acidobacteria_Gp4	incertae_sedis	incertae_sedis	*Gp4*	0.50	0.98	1.79
Acidobacteria	Acidobacteria_Gp5	incertae_sedis	incertae_sedis	*Gp5*	0.10	0.01	0.81
Acidobacteria	Acidobacteria_Gp6	incertae_sedis	incertae_sedis	*Gp6*	1.50	0.75	3.48
Acidobacteria	Acidobacteria_Gp7	incertae_sedis	incertae_sedis	*Gp7*	0.35	0.04	0.69
Acidobacteria	Acidobacteria_Gp10	incertae_sedis	incertae_sedis	*Gp10*	0.26	0.02	0.02
Acidobacteria	Acidobacteria_Gp16	incertae_sedis	incertae_sedis	*Gp16*	2.45	2.06	3.19
Acidobacteria	Acidobacteria_Gp17	incertae_sedis	incertae_sedis	*Gp17*	0.24	0.20	1.09
Acidobacteria	Acidobacteria_Gp22	incertae_sedis	incertae_sedis	*Gp22*	0.01	0.00	0.73
Actinobacteria	Actinobacteria	Acidimicrobiales	Acidimicrobiaceae	*Ilumatobacter*	1.33	3.46	2.39
Actinobacteria	Actinobacteria	Acidimicrobiales	Iamiaceae	*Iamia*	0.64	0.53	0.64
Actinobacteria	Actinobacteria	Acidimicrobiales	unclassified	unclassified	2.68	2.78	2.63
Actinobacteria	Actinobacteria	Actinomycetales	Cellulomonadaceae	*Cellulomonas*	0.03	0.50	0.11
Actinobacteria	Actinobacteria	Actinomycetales	Corynebacteriaceae	*Corynebacterium*	0.81	0.05	0.15
Actinobacteria	Actinobacteria	Actinomycetales	Geodermatophilaceae	*Blastococcus*	0.19	0.63	0.06
Actinobacteria	Actinobacteria	Actinomycetales	Intrasporangiaceae	unclassified	0.62	2.81	0.49
Actinobacteria	Actinobacteria	Actinomycetales	Kineosporiaceae	*Kineosporia*	0.56	2.59	0.43
Actinobacteria	Actinobacteria	Actinomycetales	Microbacteriaceae	*Agromyces*	0.12	0.36	0.35
Actinobacteria	Actinobacteria	Actinomycetales	Microbacteriaceae	unclassified	0.98	2.13	0.56
Actinobacteria	Actinobacteria	Actinomycetales	Micrococcaceae	*Arthrobacter*	0.92	3.37	0.90
Actinobacteria	Actinobacteria	Actinomycetales	Micromonosporaceae	unclassified	2.93	3.74	2.79
Actinobacteria	Actinobacteria	Actinomycetales	Micromonosporaceae	*Virgisporangium*	0.03	0.17	0.00
Actinobacteria	Actinobacteria	Actinomycetales	Mycobacteriaceae	*Mycobacterium*	2.04	2.16	1.80
Actinobacteria	Actinobacteria	Actinomycetales	Nakamurellaceae	*Humicoccus*	0.15	2.77	0.50
Actinobacteria	Actinobacteria	Actinomycetales	Nocardiaceae	unclassified	0.12	1.29	0.36
Actinobacteria	Actinobacteria	Actinomycetales	Nocardioidaceae	*Nocardioides*	2.82	4.66	1.59
Actinobacteria	Actinobacteria	Actinomycetales	Nocardioidaceae	unclassified	2.05	4.56	1.66
Actinobacteria	Actinobacteria	Actinomycetales	Propionibacteriaceae	*Propionibacterium*	0.46	0.17	0.32
Actinobacteria	Actinobacteria	Actinomycetales	Propionibacteriaceae	unclassified	0.24	0.51	0.28
Actinobacteria	Actinobacteria	Actinomycetales	Pseudonocardiaceae	*Actinophytocola*	0.77	0.00	0.00
Actinobacteria	Actinobacteria	Actinomycetales	Pseudonocardiaceae	*Pseudonocardia*	0.16	0.68	0.05
Actinobacteria	Actinobacteria	Actinomycetales	Pseudonocardiaceae	unclassified	0.37	0.77	0.27
Actinobacteria	Actinobacteria	Actinomycetales	Streptomycetaceae	*unclassified*	1.47	1.17	0.86
Actinobacteria	Actinobacteria	Actinomycetales	Streptosporangiaceae	*unclassified*	0.10	0.01	0.05
Actinobacteria	Actinobacteria	Actinomycetales	unclassified	unclassified	2.64	2.59	2.90
Actinobacteria	Actinobacteria	Rubrobacterales	Rubrobacteraceae	*Rubrobacter*	0.15	0.00	0.00
Actinobacteria	Actinobacteria	Solirubrobacterales	unclassified	unclassified	2.62	2.44	1.63
Actinobacteria	Actinobacteria	unclassified	unclassified	unclassified	8.55	5.71	7.68
Bacteroidetes	Bacteroidia	Bacteroidales	Porphyromonadaceae	*Dysgonomonas*	0.11	0.00	0.00
Bacteroidetes	Bacteroidia	Bacteroidales	Porphyromonadaceae	*Porphyromonas*	0.04	0.30	0.06
Bacteroidetes	Flavobacteria	Flavobacteriales	Flavobacteriaceae	*Flavobacterium*	9.24	5.77	8.09
Bacteroidetes	Flavobacteria	Flavobacteriales	Flavobacteriaceae	unclassified	6.20	0.11	0.43
Bacteroidetes	Sphingobacteria	Sphingobacteriales	Cytophagaceae	*Adhaeribacter*	0.01	2.41	0.05
Bacteroidetes	Sphingobacteria	Sphingobacteriales	Sphingobacteriaceae	*Mucilaginibacter*	0.46	0.42	0.30

Bacteroidetes	Sphingobacteria	Sphingobacteriales	Sphingobacteriaceae	*Pedobacter*	0.08	0.44	0.20
Bacteroidetes	Sphingobacteria	Sphingobacteriales	Sphingobacteriaceae	unclassified	0.15	0.00	0.02
Bacteroidetes	Sphingobacteria	Sphingobacteriales	unclassified	unclassified	0.03	0.30	0.02
Bacteroidetes	unclassified	unclassified	unclassified	unclassified	2.90	2.31	2.67
Firmicutes	Bacilli	Bacillales	Staphylococcaceae	*Staphylococcus*	0.22	0.19	0.15
Gemmatimonadetes	Gemmatimonadetes	Gemmatimonadales	Gemmatimonadaceae	*Gemmatimonas*	2.09	1.31	1.57
Nitrospira	Nitrospira	Nitrospirales	Nitrospiraceae	*Nitrospira*	0.41	0.20	0.39
Proteobacteria	Alphaproteobacteria	Rhizobiales	Hyphomicrobiaceae	*Hyphomicrobium*	0.34	0.44	0.23
Proteobacteria	Alphaproteobacteria	Rhizobiales	Hyphomicrobiaceae	unclassified	0.68	0.98	0.40
Proteobacteria	Alphaproteobacteria	Rhizobiales	unclassified	unclassified	3.13	2.41	2.49
Proteobacteria	Alphaproteobacteria	unclassified	unclassified	unclassified	0.57	0.40	0.62
Proteobacteria	Betaproteobacteria	Burkholderiales	Comamonadaceae	*Diaphorobacter*	0.33	0.04	0.01
Proteobacteria	Betaproteobacteria	unclassified	unclassified	unclassified	0.89	0.66	1.79
Proteobacteria	Deltaproteobacteria	Myxococcales	unclassified	unclassified	0.65	1.12	1.08
Proteobacteria	Deltaproteobacteria	unclassified	unclassified	unclassified	0.26	0.37	0.91
Proteobacteria	Gammaproteobacteria	Chromatiales	unclassified	unclassified	0.01	0.00	0.15
Proteobacteria	Gammaproteobacteria	Enterobacteriales	Enterobacteriaceae	*Pantoea*	0.00	0.30	0.01
Proteobacteria	Gammaproteobacteria	Enterobacteriales	Enterobacteriaceae	unclassified	1.13	0.97	0.21
Proteobacteria	Gammaproteobacteria	Pseudomonadales	Moraxellaceae	*Acinetobacter*	2.68	0.80	0.93
Proteobacteria	Gammaproteobacteria	Pseudomonadales	Moraxellaceae	*Enhydrobacter*	0.31	0.22	0.10
Proteobacteria	Gammaproteobacteria	Pseudomonadales	Pseudomonadaceae	*Pseudomonas*	1.33	0.43	0.14
Proteobacteria	Gammaproteobacteria	unclassified	unclassified	unclassified	2.03	2.42	3.09
Proteobacteria	Gammaproteobacteria	Xanthomonadales	Sinobacteraceae	*Steroidobacter*	0.35	1.24	0.47
Proteobacteria	Gammaproteobacteria	Xanthomonadales	Xanthomonadaceae	*Lysobacter*	0.10	0.68	0.37
Proteobacteria	Gammaproteobacteria	Xanthomonadales	Xanthomonadaceae	*Thermomonas*	0.00	0.02	0.56
Proteobacteria	Gammaproteobacteria	Xanthomonadales	Xanthomonadaceae	unclassified	0.62	1.05	1.58
Proteobacteria	unclassified	unclassified	unclassified	unclassified	0.81	1.23	1.11
Spirochaetes	Spirochaetes	Spirochaetales	Spirochaetaceae	*Treponema*	0.15	0.00	0.00
Verrucomicrobia	Spartobacteria	incertae_sedis	incertae_sedis	incertae_sedis	1.16	0.58	1.23
