# Supplementary material for: Long‐term ferrocyanide application via deicing salts promotes the establishment of Actinomycetales assimilating ferrocyanide‐derived carbon in soil
Source: Microb Biotechnol. 2016 May 19;9(4):502–13. doi: 10.1111/1751-7915.12362 (PMC4919992; doi:10.1111/1751-7915.12362)
Supplement: Supplementary file 6 — Table S3. Relative abundance (%) of sequences > 1% in at least one gradient fraction assigned to genus level obtained from 6 gradient fractions of DNA extracted from 13C‐labelled and unlabelled soil D, F and W based on partial 16S rRNA gene sequences after PCR amplification. Buoyant density increased among fractions 11 < 9 < 7 < 5 < 3 < 1. Genera containing 13C‐labelled OTUs are highlighted in grey. [file MBT2-9-502-s006.pdf]

Table S3

Relative abundance (%) of sequences > 1% in at least one gradient fraction assigned to genus level obtained from 6 gradient fractions of DNA extracted from ^{13}C-labelled and unlabeled soil D, F and W based on partial 16S rRNA gene sequences after PCR amplification. Buoyant density increased among fractions 11 < 9 < 7 < 5 < 3 < 1. Genera containing ^{13}C-labelled OTUs are highlighted in grey.

soil D	unlabeled						^{13}C-labelled					
Genus (1)	fract.11	fract.9	fract.7	fract.5	fract.3	fract.1	fract.11	fract.9	fract.7	fract.5	fract.3	fract.1
Gp1	1.95	0.97	0.68	0.00	0.00	0.00	1.21	2.00	1.25	1.69	2.58	0.46
Gp3	1.25	1.17	0.96	2.69	0.00	2.32	1.13	2.16	1.62	2.06	3.39	1.65
Gp4	1.53	0.15	0.25	1.45	0.00	0.70	0.88	0.40	0.22	0.08	0.36	0.01
Gp5	0.18	0.09	0.06	0.00	0.01	0.00	0.09	0.15	0.16	0.10	0.38	0.00
Gp6	1.09	2.29	1.52	0.00	0.28	2.53	1.02	2.45	1.51	1.20	1.47	2.65
Gp7	0.09	0.50	0.60	0.00	0.00	0.00	0.12	0.62	0.61	1.13	0.00	0.52
Gp10	0.04	0.21	0.15	0.96	1.05	0.00	0.10	0.23	0.18	0.00	0.23	0.00
Gp16	0.40	4.06	4.91	1.27	0.41	2.26	0.85	4.35	3.82	3.60	1.49	2.03
Gp17	0.03	0.41	0.49	0.00	0.08	0.00	0.06	0.23	0.45	0.13	0.31	0.64
Gp22	0.00	0.09	0.00	0.00	0.00	0.00	0.00	0.02	0.02	0.00	0.00	0.00
Ilumatobacter	0.78	2.29	1.43	0.00	0.61	0.00	0.65	2.22	2.44	1.58	1.78	2.15
Iamia	0.20	1.38	0.90	0.00	0.00	0.01	0.25	1.17	0.86	1.57	0.99	0.34
Acidimicrobiales_u	0.68	4.70	4.46	2.41	0.01	3.42	0.99	3.77	3.55	2.92	4.15	1.08
Cellulomonas	0.02	0.05	0.11	0.00	0.00	0.00	0.02	0.16	0.03	0.01	0.00	0.01
Corynebacterium	0.00	0.00	0.00	0.00	0.01	9.69	0.00	0.00	0.00	0.00	0.00	0.00
Blastococcus	0.04	0.24	0.50	0.00	0.00	0.00	0.09	0.23	0.35	0.31	0.09	0.47
Intrasporangiaceae_u	0.11	0.61	1.09	0.06	0.18	0.00	0.26	0.72	0.69	0.74	2.33	0.70
Kineosporia	0.13	0.50	0.87	2.62	0.00	0.00	0.10	0.43	0.45	0.00	0.00	1.60
Agromyces	0.03	0.11	0.04	0.00	0.12	0.00	0.05	0.21	0.13	0.00	0.35	0.36
Microbacteriaceae_u	0.26	0.89	1.14	0.71	0.00	3.06	0.28	0.76	0.93	0.58	2.47	0.69
Arthrobacter	0.44	0.65	0.43	0.19	0.26	0.02	1.00	1.70	1.37	2.15	1.75	1.02
Micromonosporaceae_u	0.97	3.97	5.50	2.16	0.36	1.56	0.96	3.19	4.55	3.95	3.65	4.38
Virgisporangium	0.01	0.12	0.06	0.00	0.00	0.00	0.02	0.04	0.05	0.03	0.00	0.00
Mycobacterium	0.85	2.24	2.22	5.19	0.34	0.77	0.64	1.80	1.38	2.53	1.77	4.69
Humicoccus	0.04	0.33	0.32	0.00	0.00	0.00	0.09	0.25	0.46	0.00	0.00	0.28
Nocardiaceae_u	0.07	0.08	0.21	0.00	0.00	0.00	0.05	0.11	0.15	0.00	0.03	0.79
Nocardioides	0.73	2.39	3.31	13.18	0.33	0.56	0.68	2.24	2.46	2.39	2.86	2.71
Nocardioidaceae_u	0.60	2.14	2.05	3.98	0.01	4.58	0.57	1.62	1.81	3.58	2.26	1.42
Propionibacterium	0.00	0.06	0.01	0.00	0.23	4.76	0.00	0.00	0.00	0.05	0.44	0.00
Propionibacteriaceae_u	0.04	0.39	0.56	0.00	0.00	0.00	0.15	0.27	0.27	0.44	0.69	0.00
Actinophytocola	0.18	0.70	1.46	2.93	0.00	0.16	0.22	0.62	0.57	1.20	0.77	0.44
Pseudonocardia	0.00	0.11	0.10	0.00	0.00	0.95	0.01	0.06	0.06	0.55	0.00	0.11
Pseudonocardiaceae_u	0.12	0.67	0.73	0.06	0.00	0.00	0.07	0.43	0.50	0.77	0.49	0.62
Streptomycetaceae_u	0.55	2.30	2.28	1.51	0.30	3.19	0.37	1.25	1.46	1.42	1.19	1.83
Streptosporangiaceae_u	0.01	0.06	0.01	0.00	0.00	1.09	0.00	0.00	0.02	0.00	0.00	0.00
Actinomycetales_u	0.65	4.05	3.71	1.36	0.81	2.39	0.84	3.41	3.93	4.33	3.49	2.73
Rubrobacter	0.00	0.00	0.00	1.85	0.00	0.00	0.00	0.01	0.00	0.00	0.00	0.00
Solirubrobacterales_u	0.63	4.30	5.27	0.96	0.65	0.21	0.70	3.41	4.10	5.43	3.06	2.70
Actinobacteria_u	1.98	16.28	16.08	9.38	0.56	6.78	2.45	11.18	13.31	8.58	10.09	5.89
Dysgonomonas	0.00	0.00	0.00	0.00	1.29	0.00	0.00	0.00	0.00	0.00	0.00	0.00
Porphyromonas	0.00	0.00	0.20	0.00	0.32	0.00	0.00	0.00	0.00	0.00	0.00	0.00
Flavobacterium	34.07	0.41	1.46	0.00	0.37	0.01	43.33	1.72	5.26	9.92	6.10	8.23

Flavobacteriaceae_u	2.08	0.02	0.25	0.49	67.78	1.53	1.55	0.09	0.15	0.14	0.35	0.04
Adhaeribacter	0.02	0.02	0.00	0.00	0.00	0.00	0.05	0.01	0.00	0.00	0.00	0.00
Mucilaginibacter	2.48	0.02	0.26	0.77	0.04	0.00	1.29	0.09	0.07	0.00	0.49	0.00
Pedobacter	0.34	0.02	0.00	0.00	0.00	0.00	0.31	0.03	0.25	0.00	0.00	0.00
Sphingobacteriaceae_u	0.22	0.00	0.00	1.27	0.00	0.00	0.13	0.02	0.01	0.00	0.18	0.00
Sphingobacteriales_u	0.10	0.00	0.00	0.03	0.01	0.01	0.09	0.06	0.00	0.00	0.01	0.00
Bacteroidetes_u	12.01	0.15	0.29	1.85	0.40	2.22	10.80	0.59	1.25	2.18	1.07	2.02
Staphylococcus	0.00	0.00	0.00	2.25	0.40	0.01	0.00	0.00	0.04	0.00	0.00	0.00
Gemmatimonas	1.10	2.76	2.79	0.00	0.39	2.51	0.81	3.16	2.24	2.67	2.76	3.89
Nitrospira	0.58	0.24	0.08	0.00	0.01	0.00	0.36	0.70	0.27	0.97	0.62	1.07
Hyphomicrobium	0.18	0.42	0.30	0.00	0.08	0.00	0.19	0.42	0.37	0.83	0.86	0.44
Hyphomicrobiaceae_u	0.34	1.24	0.76	0.00	0.00	0.00	0.47	1.42	1.38	0.47	1.66	0.41
Rhizobiales_u	3.04	4.70	2.53	3.33	0.44	1.17	2.44	5.77	4.02	3.11	3.17	3.80
Alphaproteobacteria_u	0.59	0.86	0.71	0.12	0.07	0.15	0.44	0.97	0.81	0.72	0.47	0.95
Diaphorobacter	0.00	0.00	0.01	0.00	3.70	0.11	0.00	0.00	0.00	0.00	0.04	0.11
Betaproteobacteria_u	0.80	1.44	0.85	0.15	0.01	0.21	0.57	1.59	1.41	0.96	1.03	1.60
Myxococcales_u	0.20	0.61	0.64	1.85	0.00	0.02	0.31	1.03	0.95	0.59	0.86	0.77
Deltaproteobacteria_u	0.65	0.23	0.13	0.00	0.00	0.23	0.55	0.50	0.28	0.22	0.02	0.36
Chromatiales_u	0.01	0.00	0.02	0.00	0.01	0.00	0.04	0.00	0.00	0.00	0.00	0.00
Pantoea	0.01	0.00	0.00	0.00	0.00	0.00	0.00	0.00	0.00	0.00	0.00	0.00
Enterobacteriaceae_u	0.01	0.00	0.01	3.52	1.29	7.02	0.01	0.00	0.03	0.31	1.01	0.36
Acinetobacter	0.00	0.00	0.15	9.07	2.52	13.87	0.02	0.00	0.01	0.17	2.74	3.58
Enhydrobacter	0.00	0.00	0.00	1.54	0.00	2.24	0.00	0.00	0.00	0.00	0.00	0.00
Pseudomonas	0.10	0.09	0.02	0.90	9.44	3.55	0.10	0.08	0.13	0.67	0.15	0.72
Gammaproteobacteria_u	3.54	1.92	1.53	1.51	0.17	0.02	2.17	2.04	1.92	2.20	3.89	3.42
Steroidobacter	0.19	0.27	0.40	0.00	0.00	0.00	0.16	0.51	0.52	1.13	0.29	0.77
Lysobacter	0.03	0.05	0.00	1.02	0.00	0.00	0.00	0.06	0.07	0.00	0.03	0.00
Thermomonas	0.00	0.02	0.00	0.00	0.01	0.00	0.01	0.00	0.00	0.00	0.00	0.00
Xanthomonadaceae_u	0.28	0.32	0.33	3.27	1.20	0.01	0.08	0.26	0.21	0.18	0.44	0.92
Proteobacteria_u	1.02	0.76	0.30	0.56	0.18	1.91	0.96	1.12	1.05	1.00	0.53	0.39
Treponema	0.00	0.00	0.00	0.00	0.00	1.79	0.00	0.00	0.00	0.00	0.00	0.00
Spartobacteria_u	3.01	0.83	0.44	0.00	0.18	0.65	2.14	2.04	1.47	0.55	0.95	1.65

soil F	unlabeled						^{13}C-labelled					
Genus (1)	fract.11	fract.9	fract.7	fract.5	fract.3	fract.1	fract.11	fract.9	fract.7	fract.5	fract.3	fract.1
Gp1	0.06	0.01	0.09	0.00	0.00	0.00	0.03	0.03	0.00	0.00	0.00	0.00
Gp3	1.48	1.04	2.20	0.80	2.31	1.03	0.98	1.80	0.67	1.25	2.45	0.72
Gp4	2.89	0.43	0.74	0.53	0.31	0.68	1.07	2.23	0.42	0.70	1.72	0.00
Gp5	0.02	0.00	0.00	0.00	0.00	0.00	0.01	0.01	0.00	0.00	0.05	0.00
Gp6	0.61	0.94	0.80	0.96	0.24	1.17	0.43	0.98	0.77	0.33	0.98	0.75
Gp7	0.04	0.03	0.00	0.00	0.00	0.14	0.01	0.01	0.01	0.14	0.05	0.00
Gp10	0.09	0.00	0.00	0.18	0.00	0.00	0.00	0.00	0.00	0.00	0.00	0.00
Gp16	0.51	4.59	2.66	2.13	2.74	1.38	1.24	1.58	3.81	1.02	1.30	1.70
Gp17	0.04	0.43	0.17	0.32	0.00	0.00	0.15	0.13	0.39	0.64	0.11	0.00
Gp22	0.00	0.00	0.00	0.00	0.00	0.00	0.00	0.00	0.00	0.00	0.00	0.00
Ilumatobacter	1.73	4.52	4.20	3.32	5.19	3.21	2.68	3.82	3.68	3.69	2.86	2.57
Iamia	0.16	0.93	0.67	0.14	0.71	0.74	0.21	0.42	1.05	1.08	0.20	0.00
Acidimicrobiales_u	0.83	4.38	3.00	4.16	2.45	2.43	1.29	2.14	4.65	2.66	2.75	2.64
Cellulomonas	0.11	0.57	0.74	0.29	0.00	0.00	0.06	0.21	0.41	0.50	0.56	2.57
Corynebacterium	0.00	0.00	0.00	0.00	0.00	0.00	0.00	0.00	0.00	0.63	0.00	0.00
Blastococcus	0.06	0.15	0.36	0.00	0.07	0.22	0.30	0.48	1.28	0.82	0.78	3.07
Intrasporangiaceae_u	0.75	2.67	1.79	3.44	1.80	4.98	1.81	2.32	3.24	3.94	2.54	4.48

Kineosporia	0.89	3.31	3.10	1.92	2.57	2.91	1.41	2.52	3.94	2.49	3.21	2.87
Agromyces	0.10	0.22	0.14	0.53	0.00	1.48	0.16	0.20	0.39	0.54	0.35	0.26
Microbacteriaceae_u	0.86	2.78	2.73	2.58	3.82	1.05	1.44	1.85	2.36	0.84	2.29	2.97
Arthrobacter	2.09	2.16	2.25	3.27	3.02	1.86	2.89	5.11	2.26	5.38	4.80	5.30
Micromonosporaceae_u	0.84	4.68	5.20	5.03	5.46	2.00	1.58	2.15	4.52	4.74	3.45	5.27
Virgisporangium	0.02	0.08	0.11	0.00	0.00	0.00	0.04	0.04	0.06	0.00	0.15	1.54
Mycobacterium	0.76	1.82	1.92	3.83	2.16	1.90	1.18	2.18	2.10	1.85	3.23	2.95
Humicoccus	0.94	3.73	3.69	3.08	3.05	0.83	1.58	2.84	3.40	5.06	2.86	2.15
Nocardiaceae_u	0.28	0.48	0.82	0.07	3.03	7.12	0.33	0.38	0.52	1.48	0.93	0.01
Nocardioides	1.38	4.67	4.99	7.02	5.61	5.96	2.75	3.69	5.89	7.92	5.27	0.78
Nocardioidaceae_u	1.24	4.36	4.41	3.87	6.92	2.58	2.32	3.52	6.45	7.52	5.91	5.57
Propionibacterium	0.00	0.00	0.00	0.39	0.45	0.37	0.00	0.00	0.01	0.36	0.00	0.49
Propionibacteriaceae_u	0.30	1.00	0.55	0.21	0.66	0.00	0.36	0.68	0.98	0.41	0.93	0.01
Actinophytocola	0.00	0.00	0.00	0.00	0.00	0.00	0.00	0.00	0.00	0.00	0.00	0.00
Pseudonocardia	0.07	0.57	0.64	0.80	2.19	1.12	0.09	0.33	0.57	1.17	0.66	0.01
Pseudonocardiaceae_u	0.12	0.62	0.55	0.17	1.68	1.70	0.38	0.46	0.76	0.79	1.99	0.01
Streptomycetaceae_u	0.46	1.50	1.92	1.40	1.27	0.50	0.71	0.52	1.70	2.22	1.75	0.09
Streptosporangiaceae_u	0.00	0.01	0.00	0.00	0.00	0.00	0.00	0.01	0.00	0.14	0.00	0.00
Actinomycetales_u	0.65	3.76	3.25	3.51	2.53	2.30	1.67	2.22	3.43	3.02	1.57	3.11
Rubrobacter	0.00	0.00	0.00	0.00	0.00	0.00	0.00	0.00	0.00	0.00	0.00	0.00
Solirubrobacterales_u	0.59	3.54	2.76	2.79	3.69	2.65	1.41	1.81	4.27	1.71	3.00	1.02
Actinobacteria_u	1.18	9.75	6.25	6.12	5.39	3.76	2.74	4.06	10.29	5.76	3.69	9.49
Dysgonomonas	0.00	0.00	0.00	0.00	0.00	0.00	0.00	0.03	0.00	0.00	0.00	0.00
Porphyromonas	0.00	0.00	0.07	0.00	0.00	0.00	0.06	0.00	0.00	0.00	0.22	3.24
Flavobacterium	23.35	0.30	3.55	3.50	3.22	1.38	21.79	5.19	0.77	0.68	1.94	3.51
Flavobacteriaceae_u	0.39	0.00	0.19	0.04	0.13	0.00	0.33	0.23	0.01	0.00	0.01	0.02
Adhaeribacter	11.30	0.24	1.36	1.02	0.83	0.00	5.97	4.68	0.10	0.27	1.96	1.17
Mucilaginibacter	1.74	0.00	0.31	1.11	0.00	0.48	0.80	0.46	0.08	0.00	0.06	0.00
Pedobacter	1.45	0.02	0.29	0.48	0.00	0.00	1.50	0.55	0.06	0.00	0.97	0.00
Sphingobacteriaceae_u	0.03	0.00	0.00	0.00	0.00	0.00	0.00	0.01	0.00	0.00	0.01	0.00
Sphingobacteriales_u	1.06	0.00	0.01	0.11	0.00	1.50	0.73	0.16	0.03	0.00	0.00	0.00
Bacteroidetes_u	8.39	0.27	0.85	3.14	0.77	2.41	7.17	1.20	0.13	1.63	1.70	0.02
Staphylococcus	0.00	0.00	0.07	0.00	0.13	1.34	0.00	0.00	0.06	0.00	0.01	0.64
Gemmatimonas	0.74	1.79	1.80	1.83	2.08	0.97	0.84	1.12	1.71	1.16	1.61	0.03
Nitrospira	0.40	0.11	0.20	0.07	0.00	0.47	0.18	0.48	0.09	0.19	0.15	0.00
Hyphomicrobium	0.41	0.66	0.58	0.20	0.00	0.91	0.36	0.67	0.53	0.07	0.87	0.00
Hyphomicrobiaceae_u	0.56	1.55	1.25	1.35	0.67	0.50	0.73	1.45	1.14	1.10	0.64	0.82
Rhizobiales_u	2.42	2.82	2.10	2.96	1.55	2.00	1.92	4.38	1.51	2.35	3.27	1.59
Alphaproteobacteria_u	0.34	0.40	0.31	0.24	0.42	0.00	0.40	0.68	0.57	0.40	0.48	0.51
Diaphorobacter	0.00	0.00	0.01	0.00	0.06	0.21	0.03	0.00	0.00	0.06	0.00	0.05
Betaproteobacteria_u	0.57	0.79	0.48	0.68	0.22	0.87	0.68	1.13	0.72	0.25	1.19	0.29
Myxococcales_u	0.68	1.33	2.11	1.21	2.18	0.63	0.58	1.00	0.99	0.54	1.00	1.16
Deltaproteobacteria_u	0.36	0.77	0.61	0.05	0.17	0.41	0.27	0.48	0.52	0.14	0.63	0.00
Chromatiales_u	0.01	0.00	0.00	0.00	0.00	0.00	0.01	0.00	0.00	0.00	0.00	0.00
Pantoea	0.00	0.00	0.00	0.00	0.00	0.00	3.52	0.03	0.00	0.00	0.00	0.00
Enterobacteriaceae_u	0.04	0.00	0.02	0.33	1.03	1.64	0.15	0.03	0.00	0.31	0.00	8.09
Acinetobacter	0.01	0.00	0.35	0.77	3.32	3.72	0.03	0.00	0.14	0.52	0.68	0.09
Enhydrobacter	0.00	0.00	0.05	0.00	0.00	2.11	0.43	0.00	0.06	0.00	0.00	0.00
Pseudomonas	0.07	0.03	0.17	0.07	0.38	0.68	0.16	0.46	0.23	0.25	0.39	2.32
Gammaproteobacteria_u	3.52	1.67	1.93	1.75	1.36	3.14	2.25	2.16	1.31	4.52	2.22	3.23
Steroidobacter	0.97	1.11	1.83	2.14	2.15	1.65	0.40	0.98	0.53	0.47	1.61	1.06
Lysobacter	0.26	0.57	1.14	0.96	0.11	1.31	0.30	0.69	0.62	0.73	1.46	0.00
Thermomonas	0.00	0.10	0.10	0.00	0.00	0.00	0.04	0.03	0.01	0.00	0.00	0.00

Xanthomonadaceae_u	0.59	1.10	0.52	0.51	1.17	2.76	0.74	1.16	0.89	1.93	0.85	0.35
Proteobacteria_u	1.70	1.19	1.50	1.29	0.95	1.63	1.05	1.75	1.12	0.97	1.13	0.55
Treponema	0.00	0.00	0.00	0.00	0.05	0.00	0.00	0.00	0.00	0.00	0.00	0.00
Spartobacteria_u	1.00	0.45	0.51	0.30	0.21	0.69	0.92	1.85	0.27	0.34	0.27	0.18

soil W	unlabeled						^{13}C-labelled					
Genus (1)	fract.11	fract.9	fract.7	fract.5	fract.3	fract.1	fract.11	fract.9	fract.7	fract.5	fract.3	fract.1
Gp1	1.00	1.20	0.76	1.30	0.75	0.90	1.75	1.34	1.24	0.09	0.50	1.29
Gp3	2.48	3.11	2.35	3.51	3.55	3.17	3.15	2.68	2.84	1.01	2.72	5.41
Gp4	4.13	1.03	0.97	2.54	1.70	2.32	3.00	0.57	0.62	1.01	1.44	2.18
Gp5	1.18	0.66	0.52	1.01	0.90	0.52	1.28	0.49	0.44	0.73	0.64	1.34
Gp6	1.72	4.36	2.87	3.36	3.08	3.77	1.73	3.41	2.25	6.42	3.31	5.47
Gp7	0.21	0.99	0.98	0.69	0.97	0.42	0.33	0.96	1.04	0.73	0.32	0.63
Gp10	0.02	0.03	0.03	0.05	0.00	0.00	0.02	0.04	0.02	0.00	0.00	0.00
Gp16	0.63	5.81	5.12	3.29	2.75	2.24	0.61	5.71	6.31	0.92	2.88	2.06
Gp17	0.27	1.48	1.64	0.79	0.55	1.20	0.24	1.49	1.29	1.28	1.21	1.68
Gp22	0.28	0.94	0.86	0.69	0.57	1.50	0.19	0.72	0.60	0.37	1.78	0.27
Ilumatobacter	0.62	2.85	2.83	2.61	2.14	2.77	1.39	3.69	4.02	1.93	1.01	2.79
Iamia	0.13	0.81	0.77	0.63	0.55	0.77	0.17	0.70	0.53	0.28	0.80	1.55
Acidimicrobiales_u	0.58	2.82	3.08	3.37	2.60	2.24	0.62	3.64	3.84	2.94	4.11	1.72
Cellulomonas	0.06	0.15	0.19	0.17	0.24	0.00	0.02	0.15	0.29	0.00	0.00	0.00
Corynebacterium	0.00	0.00	0.00	0.00	1.10	0.00	0.00	0.00	0.00	0.00	0.00	0.69
Blastococcus	0.01	0.11	0.07	0.06	0.07	0.07	0.02	0.13	0.22	0.00	0.00	0.00
Intrasporangiaceae_u	0.12	0.61	0.54	0.95	0.18	0.80	0.21	0.72	0.71	0.55	0.00	0.47
Kineosporia	0.13	0.35	0.46	0.59	0.64	0.52	0.21	0.30	0.55	0.09	1.30	0.01
Agromyces	0.00	0.18	0.21	0.30	0.24	0.27	0.02	0.15	0.31	1.74	0.62	0.11
Microbacteriaceae_u	0.22	0.71	0.66	0.89	0.84	0.95	0.14	0.55	0.67	0.46	0.00	0.65
Arthrobacter	0.16	0.53	0.31	0.59	0.88	1.32	0.23	0.70	0.36	4.86	0.37	0.53
Micromonosporaceae_u	0.76	2.41	4.07	5.49	2.71	3.14	0.62	2.92	5.53	2.29	1.53	2.01
Virgisporangium	0.01	0.00	0.00	0.00	0.00	0.00	0.00	0.02	0.02	0.00	0.00	0.00
Mycobacterium	0.42	1.10	1.13	2.15	1.50	0.77	0.45	1.28	1.11	9.36	1.83	0.47
Humicoccus	0.18	0.59	0.91	0.75	0.20	0.25	0.24	0.68	0.98	0.09	0.78	0.40
Nocardiaceae_u	0.06	0.40	0.42	1.17	0.84	0.50	0.09	0.19	0.25	0.09	0.05	0.27
Nocardioides	0.43	2.00	2.22	2.49	2.97	1.20	0.42	1.36	1.76	0.92	2.35	0.94
Nocardioidaceae_u	0.54	1.47	2.13	2.00	2.58	2.97	0.36	1.51	1.91	1.74	0.71	2.03
Propionibacterium	0.00	0.00	0.00	0.38	0.77	0.72	0.02	0.00	0.15	0.55	0.39	0.88
Propionibacteriaceae_u	0.04	0.18	0.22	0.12	0.02	0.07	0.10	0.26	0.22	0.83	0.00	1.33
Actinophytocola	0.00	0.00	0.00	0.01	0.00	0.00	0.00	0.00	0.00	0.00	0.00	0.00
Pseudonocardia	0.00	0.06	0.18	0.09	0.00	0.00	0.03	0.09	0.20	0.00	0.00	0.00
Pseudonocardiaceae_u	0.04	0.13	0.21	0.05	0.62	0.25	0.02	0.17	0.16	0.28	0.00	1.33
Streptomycetaceae_u	0.17	0.52	0.57	1.34	1.32	0.40	0.19	0.77	0.80	1.38	0.82	2.02
Streptosporangiaceae_u	0.00	0.00	0.03	0.00	0.02	0.00	0.00	0.04	0.02	0.00	0.43	0.00
Actinomycetales_u	0.50	2.79	4.08	2.99	3.19	2.54	0.76	3.60	4.00	1.28	5.89	3.21
Rubrobacter	0.00	0.00	0.00	0.00	0.00	0.00	0.00	0.00	0.00	0.00	0.00	0.00
Solirubrobacterales_u	0.31	1.93	3.20	2.30	1.56	1.75	0.29	2.03	3.42	0.64	1.12	1.00
Actinobacteria_u	1.84	11.17	12.39	8.75	8.94	8.43	1.85	10.53	10.95	7.06	5.21	5.08
Dysgonomonas	0.00	0.00	0.00	0.00	0.00	0.00	0.00	0.00	0.00	0.00	0.00	0.00
Porphyromonas	0.00	0.00	0.00	0.00	0.00	0.22	0.00	0.00	0.00	0.00	0.53	0.00
Flavobacterium	37.84	0.38	1.46	2.71	3.52	6.21	35.25	0.32	0.95	1.83	2.70	3.93
Flavobacteriaceae_u	0.69	0.01	0.12	0.09	0.44	0.27	0.68	0.02	0.00	0.00	2.33	0.53
Adhaeribacter	0.07	0.00	0.00	0.00	0.00	0.00	0.26	0.00	0.00	0.18	0.07	0.00
Mucilaginibacter	1.52	0.00	0.06	0.29	0.57	0.07	1.01	0.00	0.13	0.00	0.00	0.00

Pedobacter	0.89	0.00	0.09	0.28	0.02	0.22	0.69	0.02	0.07	0.00	0.00	0.16
Sphingobacteriaceae_u	0.10	0.00	0.00	0.01	0.00	0.00	0.10	0.00	0.00	0.00	0.00	0.00
Sphingobacteriales_u	0.08	0.00	0.01	0.00	0.00	0.02	0.07	0.00	0.02	0.00	0.00	0.05
Bacteroidetes_u	12.35	0.20	0.51	1.39	1.78	4.01	8.58	0.17	0.33	0.00	0.94	1.80
Staphylococcus	0.00	0.00	0.00	0.02	0.07	0.20	0.00	0.00	0.04	0.73	0.14	0.61
Gemmatimonas	0.40	1.51	1.85	1.73	2.71	2.22	0.52	2.17	1.40	1.01	1.16	2.16
Nitrospira	0.40	0.38	0.37	0.49	0.86	0.30	0.36	0.30	0.35	0.18	0.55	0.08
Hyphomicrobium	0.08	0.10	0.18	0.32	0.04	0.42	0.10	0.09	0.18	0.09	0.00	1.12
Hyphomicrobiaceae_u	0.13	0.53	0.40	0.50	0.33	0.57	0.35	0.60	0.55	0.73	0.00	0.05
Rhizobiales_u	1.51	3.78	2.19	2.59	1.72	2.07	2.43	3.47	2.33	2.29	2.88	2.65
Alphaproteobacteria_u	0.34	0.75	0.60	0.61	0.24	0.60	0.49	1.32	0.69	0.09	0.32	1.39
Diaphorobacter	0.00	0.00	0.00	0.00	0.02	0.00	0.00	0.00	0.04	0.00	0.02	0.01
Betaproteobacteria_u	1.03	4.22	2.58	1.36	1.41	1.87	1.91	3.37	2.00	0.37	0.50	0.90
Myxococcales_u	0.50	1.31	1.58	0.99	1.41	1.60	0.50	1.15	1.35	1.10	0.80	0.71
Deltaproteobacteria_u	0.95	0.71	0.79	0.72	0.68	0.75	1.13	0.81	1.18	1.01	1.05	1.19
Chromatiales_u	0.01	0.07	0.13	0.00	0.02	0.00	0.03	0.06	0.04	0.00	0.00	1.47
Pantoea	0.00	0.00	0.00	0.06	0.00	0.00	0.00	0.00	0.00	0.00	0.00	0.00
Enterobacteriaceae_u	0.07	0.00	0.01	0.06	0.48	0.02	0.00	0.00	0.07	0.28	0.07	1.39
Acinetobacter	0.10	0.00	0.00	0.06	0.04	0.00	0.10	0.00	0.29	0.00	10.14	0.39
Enhydrobacter	0.00	0.00	0.00	0.00	0.15	0.00	0.00	0.00	0.00	0.00	1.05	0.00
Pseudomonas	0.04	0.04	0.06	0.12	0.44	0.15	0.09	0.06	0.11	0.18	0.14	0.28
Gammaproteobacteria_u	1.55	2.43	2.23	2.66	3.99	3.39	2.34	2.30	2.27	4.68	3.47	5.76
Steroidobacter	0.12	0.47	0.24	0.04	0.75	0.55	0.16	0.36	0.49	0.28	0.41	1.72
Lysobacter	0.05	0.34	0.33	0.10	0.13	0.02	0.19	0.64	0.58	0.83	1.14	0.11
Thermomonas	0.11	0.56	0.69	0.42	0.55	0.87	0.26	0.58	0.42	0.09	1.01	1.17
Xanthomonadaceae_u	0.50	1.12	1.47	1.97	2.29	1.65	1.01	1.36	1.64	0.92	2.58	2.44
Proteobacteria_u	0.79	1.70	0.83	0.64	0.75	1.05	1.07	1.70	1.09	0.92	1.55	1.24
Treponema	0.00	0.00	0.00	0.00	0.00	0.00	0.00	0.00	0.00	0.00	0.00	0.00
Spartobacteria_u	1.49	0.82	0.74	0.87	1.21	0.87	2.20	0.77	0.91	3.30	0.66	0.95

(1) Genus or highest phylogenetic level the sequences could be assigned to
